# TRPM2 Mediates Hepatic Ischemia–Reperfusion Injury via Ca^2+^-Induced Mitochondrial Lipid Peroxidation through Increasing ALOX12 Expression

**DOI:** 10.34133/research.0159

**Published:** 2023-05-31

**Authors:** Cheng Zhong, Jing Yang, Yiyin Zhang, Xiaoxiao Fan, Yang Fan, Ning Hua, Duguang Li, Shengxi Jin, Yirun Li, Peng Chen, Yongle Chen, Xiaobo Cai, Yi Zhang, Linhua Jiang, Wei Yang, Peilin Yu, Hui Lin

**Affiliations:** ^1^ Department of General Surgery, Sir Run Run Shaw Hospital, School of Medicine, Zhejiang University, Hangzhou, P.R. China.; ^2^ Department of Biophysics and Department of Neurology of the Fourth Affiliated Hospital, Zhejiang University School of Medicine, Hangzhou 310000, P.R. China.; ^3^ Department of Physiology and Pathophysiology and Sino-UK Joint Laboratory of Brain Function and Injury of Henan Province, Xinxiang Medical University, 453003 Xinxiang, Henan, P.R. China.; ^4^ School of Biomedical Sciences, Faculty of Biological Sciences, University of Leeds, LS2 9JT Leeds, UK.; ^5^ Department of Toxicology and Department of Medical Oncology of Second Affiliated Hospital, Zhejiang University School of Medicine, Hangzhou, P.R. China.; ^6^ Zhejiang Engineering Research Center of Cognitive Healthcare, Sir Run Run Shaw Hospital, School of Medicine, Zhejiang University, Hangzhou 310020, P.R. China.; ^7^ College of Biomedical Engineering and Instrument Science, Zhejiang University, Hangzhou 310058, P.R. China.

## Abstract

Hepatic ischemia–reperfusion (IR) injury is a serious clinical problem that complicates liver resection and transplantation. Despite recent advances in understanding of the pathophysiology of hepatic IR injury, effective interventions and therapeutics are still lacking. Here, we examined the role of transient receptor potential melastatin 2 (TRPM2), a Ca^2+^-permeable, non-selective cation channel, in mediating hepatic IR injury. Our data showed that TRPM2 deficiency attenuated IR-induced liver dysfunction, inflammation, and cell death in mice. Moreover, RNA sequencing analysis indicated that TRPM2-induced IR injury occurs via ferroptosis-related pathways. Consistently, as a ferroptosis inducer, (1S,3R)-RSL3 treatment induced mitochondrial dysfunction in hepatocytes and a TRPM2 inhibitor suppressed this. Interestingly, TRPM2-mediated calcium influx caused mitochondrial calcium accumulation via the mitochondrial Ca^2+^-selective uniporter and increased the expression level of arachidonate 12-lipoxygenase (ALOX12), which results in mitochondrial lipid peroxidation during hepatic IR injury. Furthermore, hepatic IR injury-induced ferroptosis was obviously relieved by a TRPM2 inhibitor or calcium depletion, both in vitro and in vivo. Collectively, these findings demonstrate a crucial role for TRPM2-mediated ferroptosis in hepatic IR injury via increased Ca^2+^-induced ALOX12 expression, indicating that pharmacological inhibition of TRPM2 may provide an effective therapeutic strategy for hepatic IR injury-related diseases, such as during liver resection and transplantation.

## Introduction

The transient receptor potential melastatin (TRPM) family belongs to the superfamily of transient receptor potential ion channels, which includes 8 members of the TRPM subfamily in humans. The topology of TRPM channels includes 6 α-helical transmembrane-spanning domains and an intracellular N- and C-terminus, which are involved in multiple physiological and pathological processes in response to various stimuli [1]. TRPM2, the second member of the TRPM family, is a Ca^2+^-permeable cation channel that serves as a crucial oxidative stress sensor because it is activated by both reactive oxygen species (ROS) and intracellular calcium [2–4]. TRPM2 has been reported to be involved in many pathological processes, such as neurodegenerative [5,6] and vascular diseases [7] as well as tissue injury [8–10], by increasing intracellular Ca^2+^, triggering ROS production, and inducing inflammation. It is also known to play a detrimental role in ischemia–reperfusion (IR) injury in many types of tissues, such as the liver [11], kidney [12], and brain [13–15]. However, the role of TRPM2 in myocardial IR injury remains controversial. There is evidence that TRPM2-mediated neutrophil activation and accumulation in response to myocardial IR leads to myocardial infarction [16]. In contrast, other studies have shown that TRPM2 protected the myocardium from IR injury by maintaining mitochondrial function and reducing ROS production [17,18]. Although recent studies have shown that inhibition of TRPM2 reduces hepatic IR injury in mice [19,20], the underlying mechanism remains unclear.

Hepatic IR injury is a major complication of liver resection and transplantation. It can induce a severe inflammatory response that exacerbates liver injury after surgery and aggravates rejection and failure following liver transplantation [21,22]. However, currently, there is no pharmacological intervention protecting the liver from hepatic IR injury. Previous studies have revealed that IR injury is triggered by energy depletion in the mitochondria during ischemia, intracellular Ca^2+^ overloading, elevated ROS generation, metabolic disorders, and activation of inflammatory cascades during reperfusion [7]. Clinical evidence suggests that the preoperative serum ferritin level of the donor is an independent risk factor for hepatic IR during liver transplantation [23]. Further in vivo studies indicated that liver IR injury was significantly reduced by the ferroptosis inhibitor, ferrostatin-1 (Fer-1), or α-tocopherol, and exacerbated by iron overload [23,24]. These studies suggest that ferroptosis plays an important role in the pathogenesis of hepatic IR injury.

Ferroptosis is a ROS-dependent form of cell death, and the main features were iron accumulation and lipid peroxidation [25,26]. Ferroptotic cells display mitochondrial abnormalities characterized by enhanced condensation and membrane density [27]. Lipid oxidation during ferroptosis can be induced by accumulated, iron-triggered, non-enzymatic Fenton reactions and enzymatic reaction pathways mainly promoted by arachidonate lipoxygenases (ALOXs) [28]. Additionally, inhibition of components of the antioxidant system, such as nuclear factor erythroid 2-related factor 2 (Nrf2) or glutathione peroxidase 4 (GPX4) or degradation of Ferritin, also enhanced the generation of lipid peroxidation [29–31]. Ferroptosis reportedly participates in IR injury in the brain, heart, kidney, intestines, and liver [32–36]. For instance, acyl-CoA synthetase long-chain family member 4 (ACSL4)-mediated ferroptosis is involved in kidney IR injury [37]. Reduced GPX activity, accumulation of iron, and lipid peroxidation are essential components that cause heart IR injury through triggering ferroptosis [38]. Moreover, inhibition of iron transport by HUWE1-induced transferrin receptor degradation could suppress ferroptosis during hepatic IR [36], and inhibition of ferroptosis using Fer-1 can strongly alleviate heart IR injury in mice [33]. A recent study reported iron overloading as an independent risk factor in patients undergoing liver transplantation, suggesting that ferroptosis may be involved in the pathogenesis of hepatic IR injury [23]. To date, the mechanism underlying ferroptosis and hepatic IR injury remains elusive.

In this study, we investigated the role of TRPM2 in hepatic IR injury, both in vitro and in vivo. RNA sequencing analysis revealed that ferroptosis-related genes are enriched in TRPM2-induced IR injury. Our findings provide the first insight into how TRPM2 mediates hepatic IR injury via induction of mitochondrial lipid peroxidation and ferroptosis. Notably, we verified the protective effect of the TRPM2-specific inhibitor, A10, during hepatic IR injury in vitro and in vivo. This provided strong evidence that TRPM2 inhibitors might be potential drug candidates for preventing hepatic IR injury during liver resection and transplantation.

## Results

### TRPM2 knockout ameliorates hepatic IR injury

**Fig. 1.**
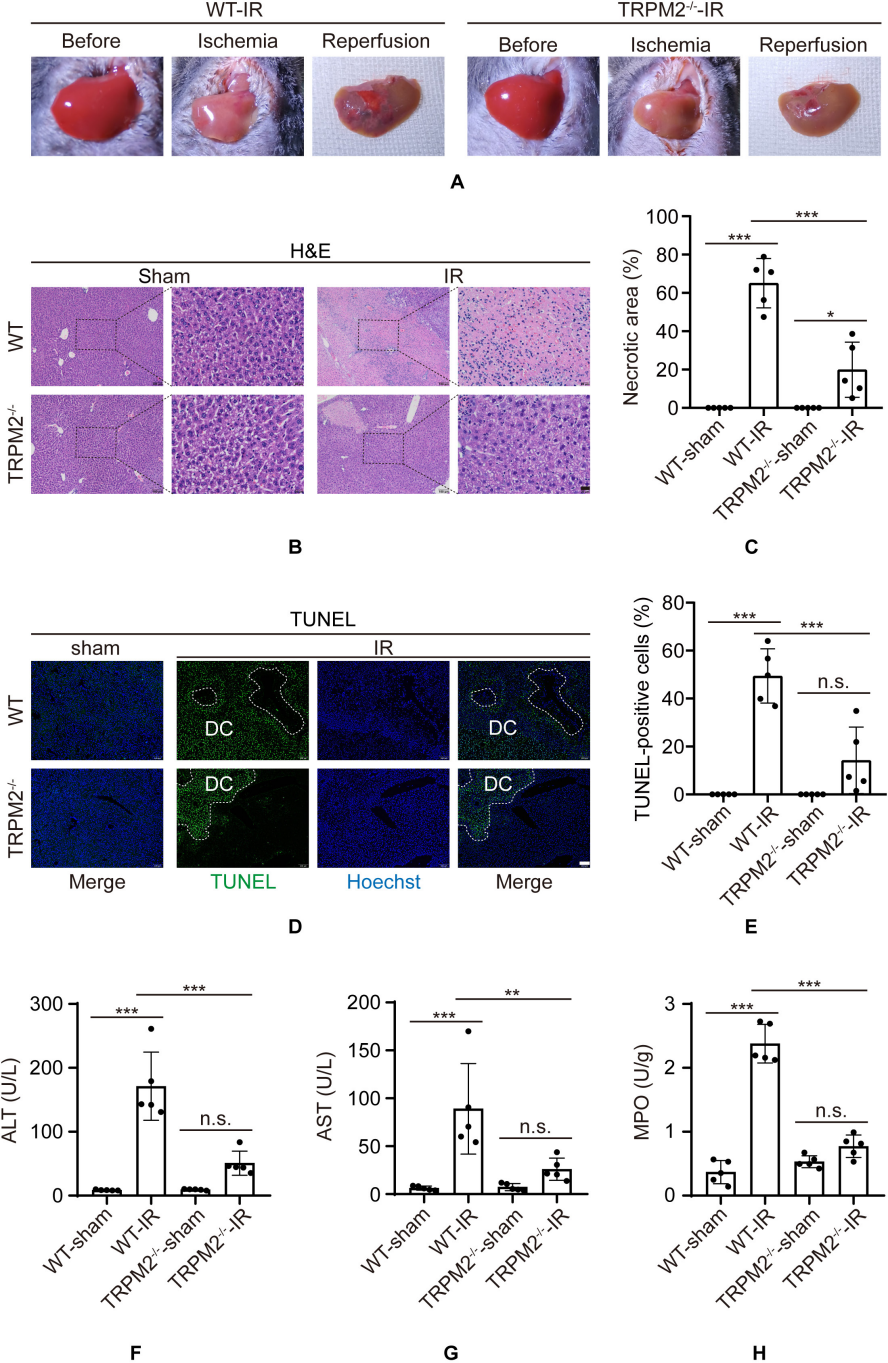
Effects of *TRPM2* knockout on hepatic ischemia–reperfusion injury. (A) TRPM2^−/−^ and wild-type mice were subjected to ischemia followed by reperfusion. The control mice in each group did not undergo ischemia. Gross morphology of the liver is shown at various time points. (B and C) Hematoxylin and eosin stain images show histopathological changes, and the necrotic area was quantified. Scale bars: 100 μm and 20 μm. (D and E) TUNEL staining was conducted to identify dying cells (DC). Dying cells appear green and the nuclei appear blue. Scale bar: 100 μm. (F to H) Detection of serum alanine aminotransferase, aspartate aminotransferase, and myeloperoxidase activity levels of liver samples. Each group included five mice. All results are expressed as mean ± standard deviation. n.s. = no significance, *P < 0.05, **P < 0.01, ***P < 0.001. IR, ischemia–reperfusion.

Recently, hepatic IR injury was observed to decrease in TRPM2 knockout mice or by inhibition through TRPM2 siRNA [19,20]. To validate the function of TRPM2 during hepatic IR in vivo, a mouse model of partial liver IR injury was used. Both wild-type (WT) and TRPM2^–/–^ mice were subjected to 1 h of ischemia, and sacrificed after 3-, 6-, 12-, 24-, and 48-h reperfusion (Fig. S1A and B) [39]. The real-time polymerase chain reaction (PCR) analysis exhibited increased TRPM2 mRNA expression from reperfusion for 12 to 48 h. Then, 24-h reperfusion was chosen to further detect TRPM2 function (Fig. S1C). Gross hepatic IR injury in TRPM2^–/–^ mice was lower than that in WT mice (Fig. 1A). The degree of IR-induced liver damage, including lobular hemorrhage, congestion, cellular swelling, and necrosis, was revealed by hematoxylin and eosin (H&E) staining (Fig. 1B and C), and cell death was identified by terminal deoxynucleotidyl transferase-mediated dUTP nick-end labeling (TUNEL) staining (Fig. 1D and E). Data showed that both were less severe in TRPM2^–/–^ mice than those in WT mice. The levels of 2 established markers of liver injury, alanine aminotransferase (ALT) and aspartate aminotransferase (AST), were significantly decreased in TRPM2^–/–^ mice with hepatic IR (Fig. 1F and G). A recent study reported that the local innate inflammatory response driven by macrophages, platelets, and neutrophils is the primary cause of hepatocyte damage during IR [40]. Hepatic IR-induced neutrophil infiltration, which is considered a reliable marker for neutrophil activity [41], was decreased in TRPM2^–/–^ mice compared with that in WT mice, with a level similar to that in mice without exposure to IR (Fig. 1H). Furthermore, histologically detected liver damage and function were similar between the TRPM2^–/–^ and WT mice that underwent sham surgery. In addition, immunostaining showed enhanced expression of TRPM2 in liver tissues subjected to IR (Fig. S1D).

### Ferroptosis-related genes are downregulated in TRPM2^–/–^ mice exposed to hepatic IR

**Fig. 2.**
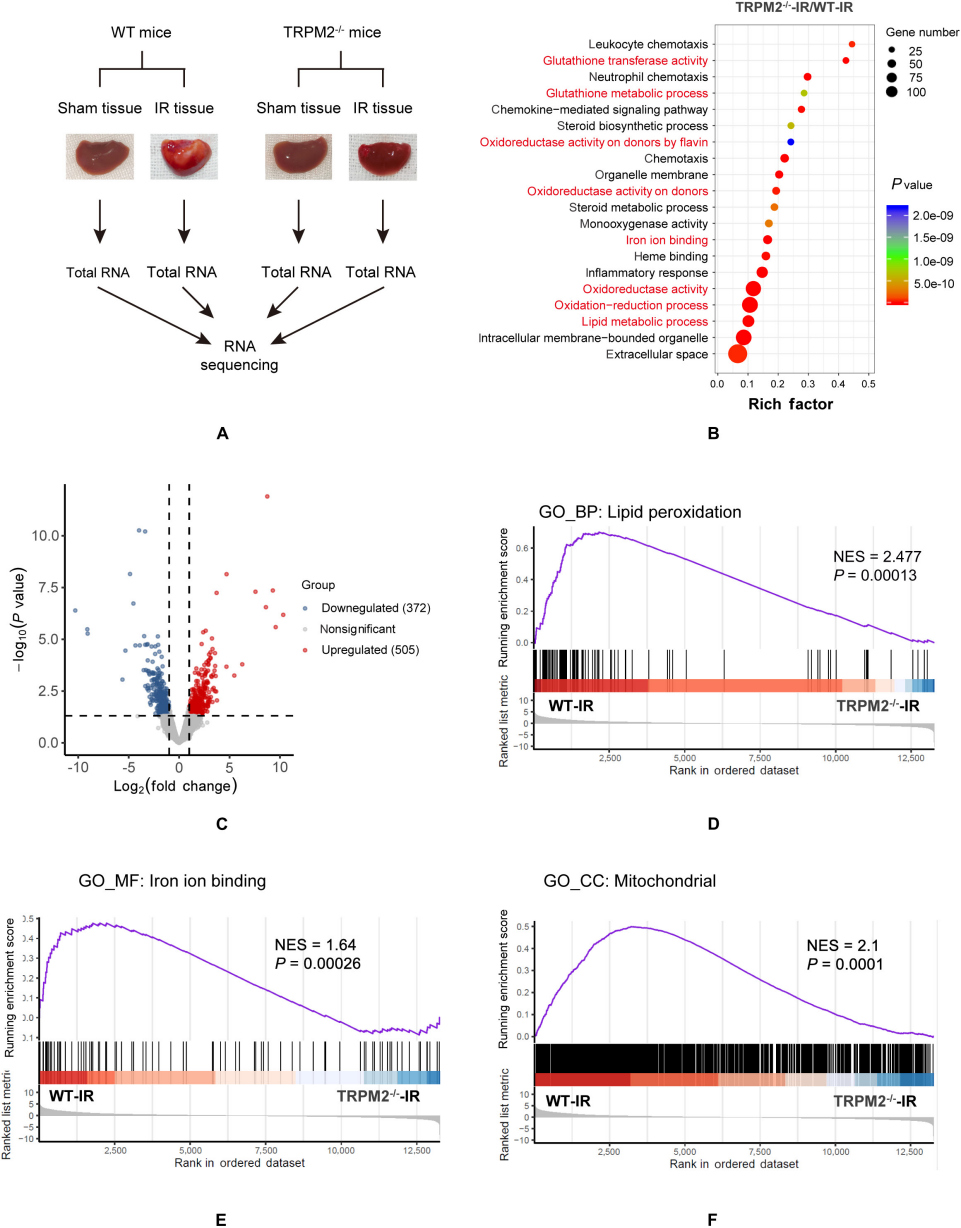
Transcriptome analysis of liver tissues from TRPM2^−/−^ and wild-type mice. (A) Workflow of the transcriptome analysis. (B) A volcano plot showing upregulated (blue) and downregulated (orange) genes in the liver tissue of TRPM2^−/−^ mice subjected to hepatic ischemia–reperfusion (IR) compared with that of wild-type (WT) mice. (C) Gene ontology pathway enrichment analysis of differentially expressed genes generated from TRPM2^−/−^ and WT mice that underwent IR. (D to F) Gene Set Enrichment Analysis of lipid peroxidation, iron ion binding, and mitochondria-associated pathway generated from TRPM2^−/−^ and WT mice that underwent IR.

To explore the potential mechanism by which TRPM2 mediates hepatic IR injury, we performed transcriptomic sequencing of liver tissues from WT and TRPM2^–/–^ mice with or without exposure to IR (Fig. 2A). RNA sequencing and gene ontology (GO) pathway enrichment analyses showed that most differentially expressed genes in the TRPM2^–/–^-IR mice compared with that of the WT-IR mice were enriched in the pathways associated with ferroptosis, including redox regulation, glutathione (GSH) metabolic process, lipid metabolism, and iron ion binding (Fig. 2B and C). Differentially expressed genes in the TRPM2^–/–^-sham mice were mainly clustered in the biosynthetic process compared with that of the WT-sham mice (Fig. S2A). We further compared the transcriptional changes elicited by TRPM2 deficiency and IR using Gene Set Enrichment Analysis (GSEA), which enabled the detection of statistically significant differences in biological pathways by comparing datasets available from predefined gene lists. GSEA enrichment plots revealed that ferroptosis-related pathways, including lipid peroxidation, iron ion binding, and GSH metabolism, were significantly upregulated in WT mice subjected to IR injury compared with those in TRPM2^–/–^ mice (Fig. 2D and E and Fig. S2B), and the mRNA of genes attributed to lipid peroxidation, such as ALOXE3, ACSL1, ACSL3, COX2, and CYP1A2, was also upregulated in WT mice under hepatic IR (Fig. S2C). Moreover, GSEA showed that the mitochondria-associated pathway was enriched (Fig. 2F).

### TRPM2 inhibition attenuated ferroptosis in hepatic injury induced by IR in vivo and oxygen and glucose deprivation/reperfusion in vitro

**Fig. 3.**
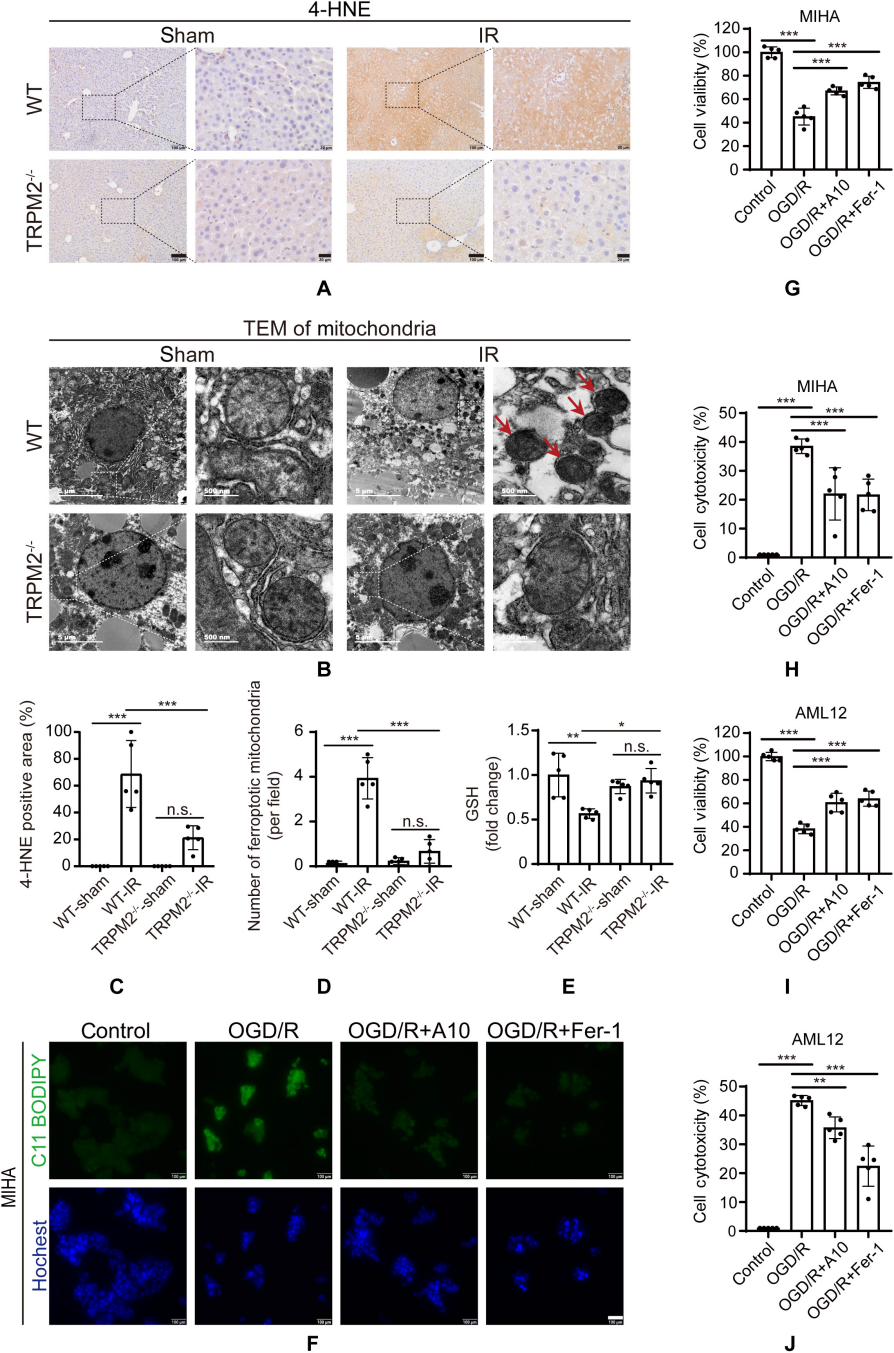
Inhibition of TRPM2 reduces hepatic ischemia–reperfusion injury via ferroptosis. (A and C) Representative images showing (A) 4-hydroxy-2-nonenal (4-HNE) staining and (C) quantification of the 4-HNE levels in liver sections of TRPM2^−/−^ and wild-type (WT) mice without or with exposure to ischemia–reperfusion (IR). Scale bars: 100 μm and 20 μm. (B) Representative transmission electron micrographs showing ferroptosis in hepatic tissues of TRPM2^−/−^ and WT mice without or with exposure to IR. Red arrows highlight shrunken mitochondria. Scale bars: 5 μm and 500 nm. (D) Mean numbers of shrunken mitochondria derived from micrographs as shown in panel B. (E) Mean glutathione levels in hepatic tissues of TRPM2^−/−^ and WT mice without or with exposure to IR. Each group included five mice. (F) Representative C11 BODIPY 581/591 staining (green) images showing the lipid peroxidation levels in MIHA liver cells without or with exposure to oxygen and glucose deprivation/reperfusion (OGD/R). Cells were counterstained with Hoechst (blue). Scale bar: 100 μm. (G and I) Summary of cell viability examined using a cell counting kit-8 in MIHA and AML12 cells under indicated conditions. (H and J) Summary of cell cytotoxicity in MIHA and AML12 cells examined by detecting LDH release. All results are shown as mean ± standard deviation. n.s. = no significance, *P < 0.05, **P < 0.01, ***P < 0.001. IR, ischemia–reperfusion; TEM, transmission electron microscopy; OGD/R, oxygen and glucose deprivation/reperfusion.

We next examined the role of ferroptosis in mediating hepatic IR injury. Excessive lipid peroxidation is one of the hallmarks of ferroptosis and is mainly driven by oxidative stress due to the overproduction of ROS and/or suppression of the antioxidant capacity of cells. Increased levels of 4-hydroxy-2-nonenal (4-HNE), a metabolite derived from lipid peroxidation, and reduced levels of GSH, which is crucial for the antioxidant defense mechanism, are recognized as ferroptotic markers [29]. Exposure of WT mice to IR induced a massive increase in 4-HNE levels in hepatic tissues and were markedly decreased in TRPM2^–/–^ mice (Fig. 3A and C). Exposure to IR also altered the morphological features of mitochondria in hepatic tissues. Transmission electron microscopy (TEM) showed that the mitochondria in IR-exposed WT mice were smaller in size and contained fewer cristae, and these changes were noticeably mitigated in TRPM2^–/–^ mice (Fig. 3B and D). Furthermore, GSH levels were reduced in WT mice after IR and no significant changes in TRPM2^–/–^ mice (Fig. 3E). These results suggest that ferroptosis might be involved in TRPM2-induced hepatic IR.

We also investigated the role of TRPM2 and ferroptosis in mediating hepatic IR injury using MIHA and AML12 liver cells, as well as oxygen and glucose deprivation/reperfusion (OGD/R), an in vitro model of IR. Cells subjected to OGD/R showed significantly enhanced mRNA and protein expression of TRPM2, especially at reperfusion for 12 and 24 h (Fig. S3A to C). In this experiment, the TRPM2 inhibitor, A10 (chemical name: 6-bromo-8-methyl-2-(3-phenyl-1H-pyrazol-4-yl)-2,3-dihydroquinazolin-4(1H)-one), was used. This inhibitor was designed and synthesized by our group and it specifically binds to the NUDT9-H domain of TRPM2 to block TRPM2-induced Ca^2+^ influx, and has no effect on other subtypes of the TRPM channels measured in our previous study [42]. In both cell types, exposure to OGD/R enhanced lipid peroxidation and was strongly reduced after treatment with A10, a TRPM2-specific inhibitor [42], and Ferrostatin-1 (hereinafter referred to as Fer-1), a well-known ferroptosis inhibitor [27] (Fig. 3F and Fig. S3D to F). Exposure to OGD/R decreased cell viability and enhanced LDH release, and both were suppressed after treatment with A10 or Fer-1 (Fig. 3G to J). Collectively, these in vitro results strongly indicate that OGD/R induced cytotoxicity in liver cells via ferroptosis, and thus were highly consistent with the above-described in vivo findings.

### TRPM2 inhibition alleviates RSL3-induced ferroptosis

**Fig. 4.**
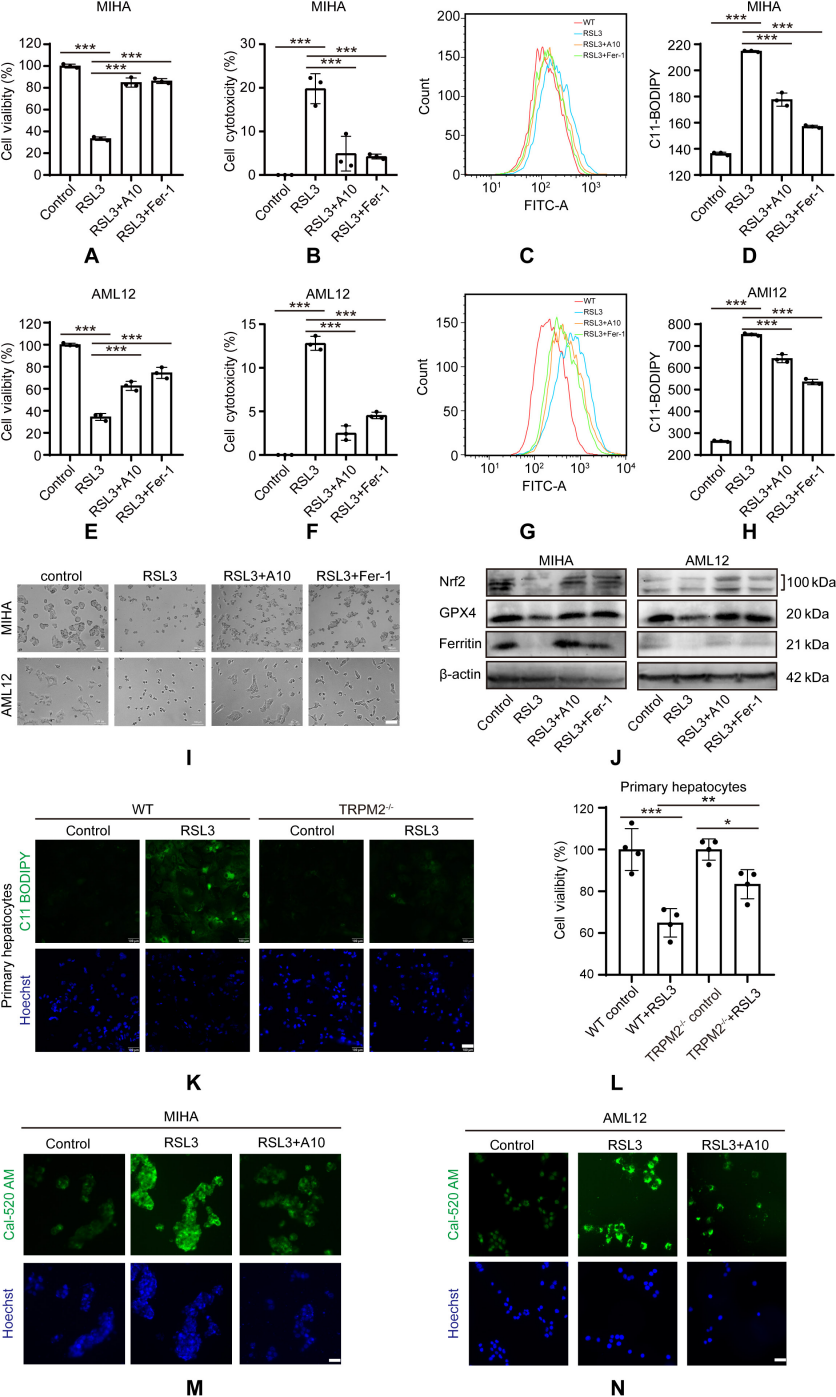
Inhibition of TRPM2 blocked RSL3-induced ferroptosis. (A, B, E, and F) Cell viability and cytotoxicity in MIHA and AML12 cells under control conditions after exposure to RSL3 without or with pretreatment with A10 or ferrostatin-1. (C, D, G, and H) Flow cytometry analysis of lipid peroxidation using C11 BODIPY 581/591 in (C and D) MIHA and (G and H) AML12 cells under indicated conditions. (I) Representative images showing changes in cell morphology. Scale bar: 100 μm. (J) Representative Western blots showing Nrf2, GPX4, and Ferritin protein expressions in MIHA and AML12 cells under indicated conditions. (K) Representative C11 BODIPY 581/591 staining (green) images showing the lipid peroxidation levels in primary hepatocytes from wild-type and TRPM2^−/−^ mice under indicated conditions. Cells were counterstained with Hoechst (blue). Scale bar: 100 μm. (L) Cell viability of primary hepatocytes under indicated conditions. (M and N) Representative Cal-520 AM (green) staining images showing intracellular Ca^2+^ in MIHA and AML12 cells under indicated conditions. Cells were counterstained with Hoechst (blue). Scale bar: 200 μm. All results are shown as mean ± standard deviation. *P < 0.05, **P < 0.01, ***P < 0.001. Fer-1, ferrostatin-1.

Next, we explored the role of TRPM2 in mediating ferroptosis following treatment with (1S,3R)-RSL3 (hereinafter referred to as RSL3), an inducer of ferroptosis, in MIHA and AML12 cells. Expectedly, exposure to RSL3 significantly reduced cell viability and increased cell cytotoxicity, and both were inhibited by pretreatment with A10 or Fer-1 (Fig. 4A, B, E, F, and I). Moreover, RSL3 treatment enhanced lipid peroxidation and was reduced by pretreatment with A10 and Fer-1 in MIHA and AML12 cells (Fig. 4C, D, G, and H). We further found that A10 and Fer-1 rescued the reduction in GPX4, Nrf2, and Ferritin expression levels after RSL3 treatment in MIHA and AML12 cells (Fig. 4J and Fig. S4A to C), supporting that TRPM2 is critical in RSL3-induced ferroptosis. To further corroborate the relationship between TRPM2 and ferroptosis, we detected the expression levels of key genes that contribute to ferroptosis, including ChaC GSH-specific gamma-glutamylcyclotransferase 1 (*CHAC1*), isoforms of the lipoxygenase family (*ALOX12* and *ALOX15*), and prostaglandin endoperoxide synthase 2 (*PTGS2*) in RSL3-treated cells. Our data consistently showed that the upregulated expression of these genes was also inhibited by pretreatment with A10 and Fer-1 (Fig. S4D and E). We performed further experiments on primary hepatocytes isolated from WT and TRPM2^–/–^ mice. Exposure to RSL3 significantly increased the level of lipid peroxidation in the hepatocytes of WT mice, whereas RSL3-induced lipid peroxidation was significantly lower in those of TRPM2^–/–^ mice (Fig. 4K and Fig. S4F). Moreover, exposure to RSL3 reduced the viability of hepatocytes in WT mice, and the cytotoxic effect was alleviated in those of TRPM2^–/–^ mice (Fig. 4L).

Given that TRPM2 regulates diverse pathophysiological processes via promotion of extracellular Ca^2+^ influx [43], we wondered whether TRPM2-mediated Ca^2+^ influx is involved in ferroptosis. In both MIHA and AML12 cells, exposure to RSL3 led to an increase in intracellular Ca^2+^ levels as detected by Cal-520 AM [44], and this was inhibited by treatment with A10, supporting that TRPM2 is responsible for RSL3-induced intracellular Ca^2+^ signaling during ferroptosis (Fig. 4M and N and Fig. S4G and H).

### TRPM2 inhibition alleviates ferroptosis through reducing mitochondrial Ca^2+^ accumulation and lipid peroxidation

**Fig. 5.**
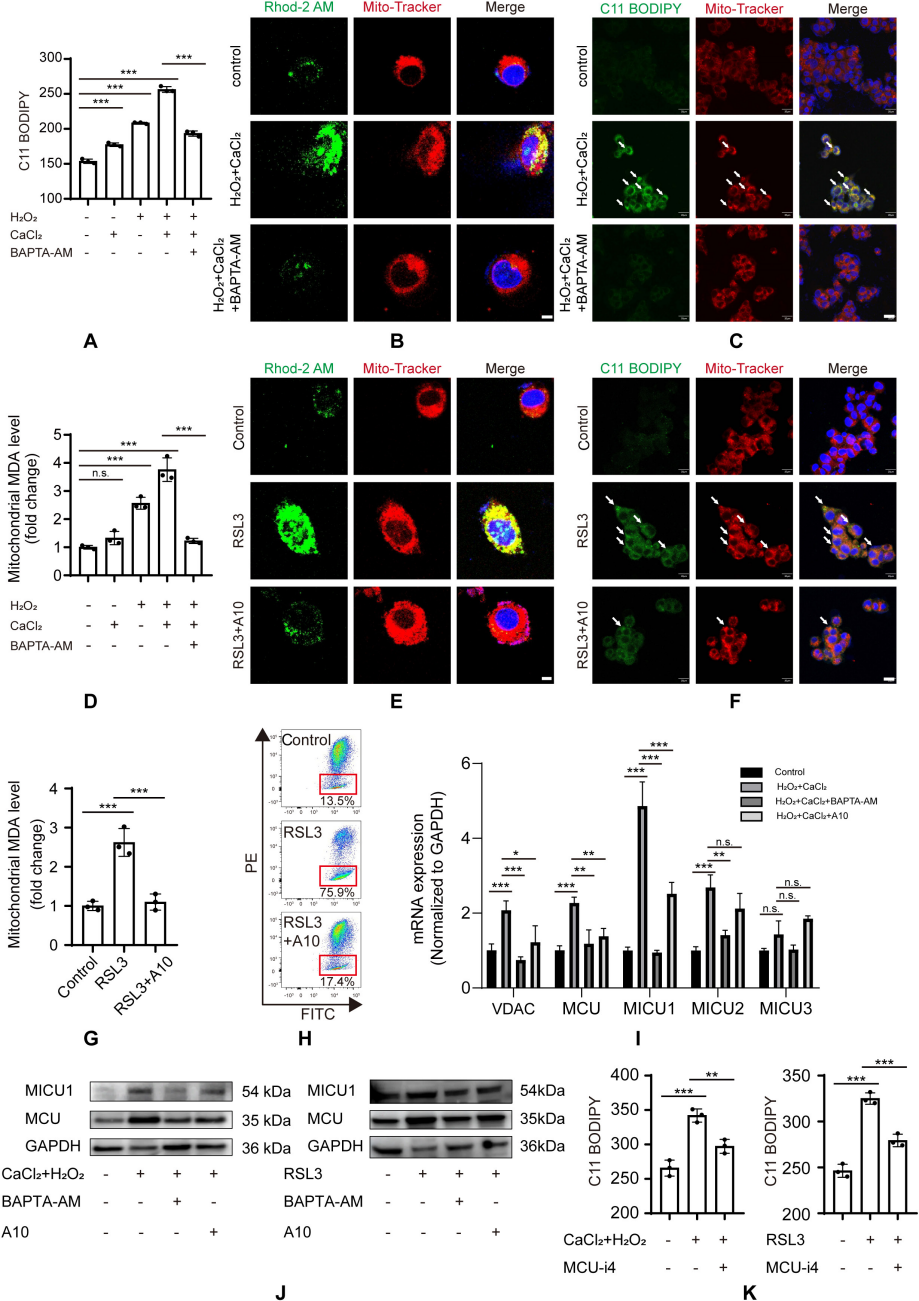
Calcium influx promotes mitochondrial lipid peroxidation in MIHA cells. (A) Flow cytometry of lipid peroxidation using C11 BODIPY 581/591 in cells under indicated conditions. (B) Representative confocal micrographs of mitochondrial Ca^2+^ influx and mitochondria cells using Rhod-2 AM (green) and MitoTracker (red) staining, respectively. Cells were counterstained with Hoechst (blue). Scale bar: 5 μm. (C) Representative confocal micrographs showing lipid peroxidation and mitochondria with C11 BODIPY (green) and MitoTracker (red) staining, respectively. Cells were counterstained with Hoechst (blue). Scale bar: 20 μm. (D) Malondialdehyde (MDA) levels were detected by an MDA assay kit under indicated conditions. (E) Representative confocal micrographs of mitochondrial Ca^2+^ influx during RSL3 treatment. Scale bar: 5 μm. (F) Representative confocal micrographs showing lipid peroxidation under RSL3 treatment. Scale bar: 20 μm. (G) MDA levels were detected by an MDA assay kit during RSL3 treatment. (H) Flow cytometry of mitochondrial membrane potential using JC-1 staining. (I) The mRNA expression of mitochondrial Ca^2+^ transporter related genes. (J) Representative Western blots showing mitochondrial Ca^2+^-selective uniporter expression under indicated conditions. (K) Flow cytometry of lipid peroxidation under indicated conditions. All results are shown as mean ± standard deviation. n.s. = no significance, *P < 0.05, **P < 0.01, ***P < 0.001.

As demonstrated, TRPM2-induced Ca^2+^ influx plays a crucial role in ferroptosis. We then further addressed the mechanism by which Ca^2+^ regulates ferroptosis. A low concentration of H_2_O_2_ was used to activate TRPM2 [45,46], and increased intracellular Ca^2+^ was observed when cells were exposed to the synchronous application of H_2_O_2_ and extracellular Ca^2+^ (Fig. S5A). As it is well known that lipid peroxidation is the hallmark of ferroptosis, we detected the effect of Ca^2+^ overload on ferroptosis by using C11 BODIPY 581/591. According to the results of flow cytometry analysis and fluorescence microscopy, H_2_O_2_ induced modest lipid peroxidation, was strongly elevated by the addition of extracellular Ca^2+^, and could be prevented by pretreatment with BAPTA-AM (Fig. 5A and Fig. S5B and C). These results supported that TRPM2-induced Ca^2+^ overload accelerates lipid peroxidation. Interestingly, H_2_O_2_-induced intracellular Ca^2+^ overload was highly localized in the mitochondria (Fig. S5D), suggesting that TRPM2-induced Ca^2+^ overload resulted in Ca^2+^ accumulation in the mitochondria. To confirm this result, mitochondrial Ca^2+^ uptake was detected using the fluorescent dye, Rhod-2 AM [47–49]. Consistently, mitochondrial Ca^2+^ increased after activating TRPM2 after H_2_O_2_ and CaCl_2_ treatment but decreased significantly when treated with BAPTA-AM (Fig. 5B and Fig. S5E and F). Furthermore, the activation of TRPM2 by exposure to H_2_O_2_ increased mitochondrial lipid peroxidation and malondialdehyde (MDA) levels in the presence of extracellular Ca^2+^, and this was inhibited after BAPTA-AM treatment (Fig. 5C and D). These data suggest that TRPM2 mediates increased intracellular Ca^2+^ accumulation in mitochondria and triggers mitochondrial lipid peroxidation.

We further determined whether TRPM2 is involved in the accumulation of mitochondrial Ca^2+^ and lipid peroxidation induced by RSL3. Our data showed that RSL3 treatment strongly induced mitochondrial Ca^2+^ accumulation and was suppressed after A10 pretreatment (Fig. 5E and Fig. S5G and H). Moreover, A10 treatment also inhibited RSL3-induced mitochondrial lipid peroxidation in MIHA cells and MDA levels in mitochondria (Fig. 5F and G). Moreover, we measured mitochondrial function using JC-1 and found that this was disrupted by RSL3 and after pretreatment with A10 (Fig. 5H and Fig. S5I). These data support the hypothesis that TRPM2 plays a key role in RSL3-induced mitochondrial calcium accumulation and lipid peroxidation.

### Increased cytoplasmic calcium transport to mitochondria via the mitochondrial Ca^2+^-selective uniporter complex

A previous study reported that mitochondria play a crucial role in cytoplasmic calcium balance. Increased intracellular calcium transported to mitochondria mainly depends on voltage-dependent anion channel 1 (VDAC1) of the outer mitochondrial membrane and the mitochondrial Ca^2+^-selective uniporter (MCU) complex of the inner mitochondrial membrane. Calcium transport to mitochondria via the MCU complex is a rate-limiting step [50]. Three Ca^2+^-regulatory subunits, MICU1, MICU2, and MICU3, and MCU in the intermembrane space were identified as components of the MCU complex [51]. MICU1 acts as the gatekeeper by setting a threshold to inhibit Ca^2+^ uptake when cytosolic Ca^2+^ concentrations are low [52,53]. Our data showed increased mRNA expression of *VDAC1*, *MCU*, *MICU1*, and *MICU2* when calcium overload occurred, whereas the decreased cytoplasmic calcium concentrations after BAPTA-AM or A10 suppressed their mRNA expression levels (Fig. 5I). We further found that MCU and MICU1 expression markedly increased after calcium overload or RSL3 treatment and decreased after pretreatment with BAPTA-AM or A10 (Fig. 5J and Fig. S5J). Considering the essential role of MICU1 in calcium transport into mitochondria, the MICU1 inhibitor, MCU-i4, was used to suppress the MCU complex to further verify its function during calcium overload and ferroptosis [54]. Lipid peroxidation was visibly suppressed when cells were incubated with MCU-i4 before exposure to calcium overload or RSL3 (Fig. 5K). These data showed that the increased intracellular calcium induced by TRPM2 was transported to mitochondria by the MCU complex.

### Ca^2+^ promotes ALOX12 to induce mitochondrial lipid peroxidation

**Fig. 6.**
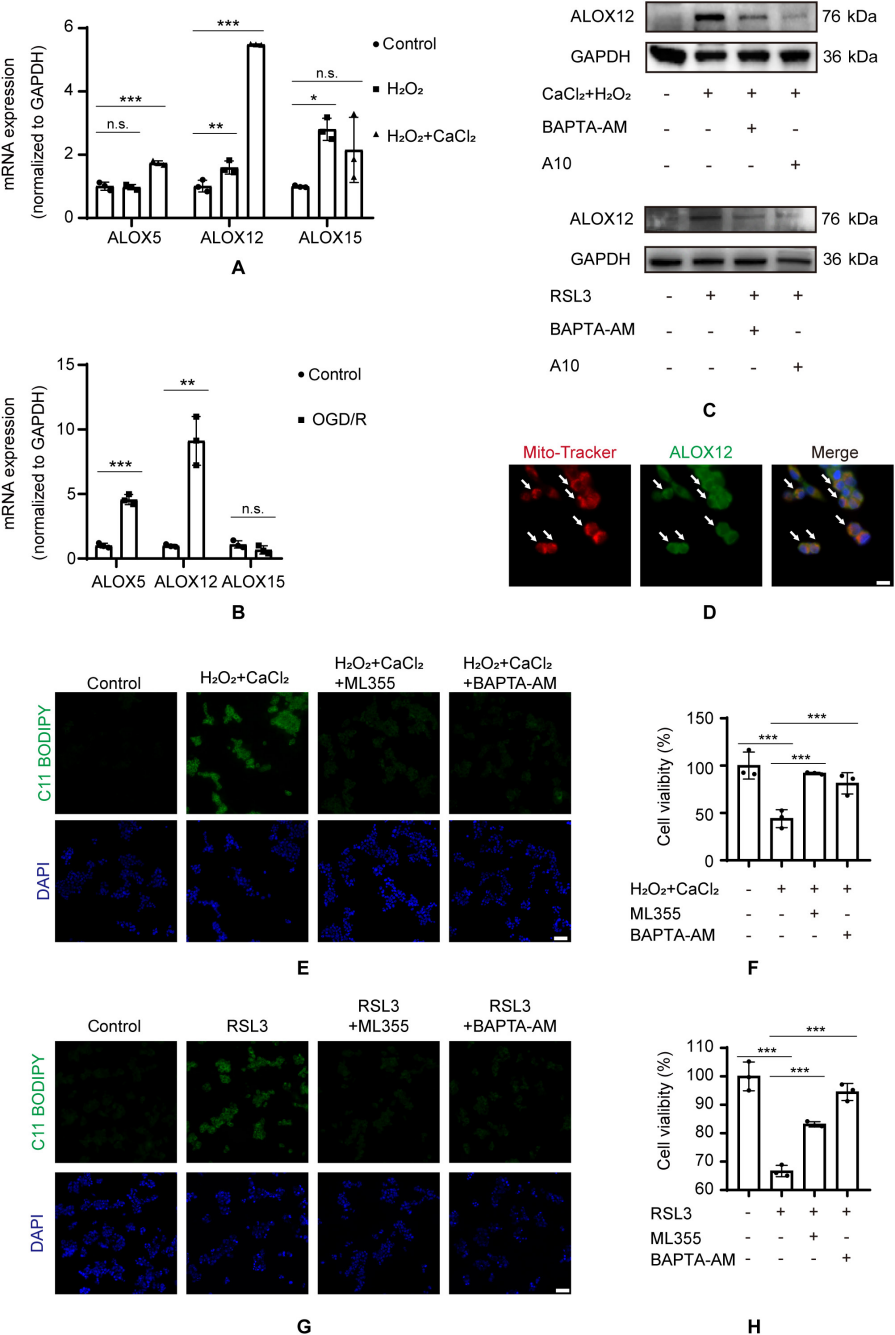
Calcium influx activated ALOX12 to promote mitochondrial lipid peroxidation. (A and B) The mRNA expression of *ALOX5*, *ALOX12*, and *ALOX15* after CaCl_2_ + H_2_O_2_ or oxygen and glucose deprivation/reperfusion stimulation. (C) Representative Western blots showing ALOX12 expression under indicated conditions. (D) Representative confocal micrographs showing the localization of ALOX12 using immunofluorescence (green) and mitochondria with MitoTracker (red) staining. Cells were counterstained with Hoechst (blue). Scale bar: 10 μm. (E and G) Lipid peroxidation and (F and H) cell viability of MIHA cells after exposure to CaCl_2_ + H_2_O_2_ or RSL3 without or with ML355 or BAPTA-AM pretreatment. Scale bar: 100 μm. n.s., no significance, *P < 0.05, **P < 0.01, ***P < 0.001.

Based on the findings in the Increased cytoplasmic calcium transport to mitochondria via the mitochondrial Ca^2+^-selective uniporter complex section, we found that TRPM2-mediated Ca^2+^ influx accumulated in the mitochondria, inducing mitochondrial lipid peroxidation. We further investigated the mechanism underlying this process. Previous studies have highlighted that ALOXs enzymatically initiate mediators of phospholipid peroxidation during ferroptosis [55–57], and ALOX5, ALOX12 activity, and ALOX15 translocation to the mitochondria could be directly mediated by Ca^2+^ [58–60]. Therefore, we explored whether TRPM2-mediated Ca^2+^ influx activates ALOXs to promote lipid peroxidation. We primarily studied the mRNA expression of *ALOX5*, *ALOX12*, and *ALOX15* during TRPM2-mediated calcium overload and OGD/R-activated cells, and found that *ALOX12* expression obviously increased (Fig. 6A and B). We further found that *ALOX12* expression was markedly increased during calcium influx after treatment with CaCl_2_ and H_2_O_2_, whereas this was suppressed when treated with A10 or BAPTA-AM (Fig. 6C and Fig. S6A). As shown in Fig. 5, TRPM2-induced calcium overload promoted lipid peroxidation mainly in the mitochondria. Next, we detected the location of ALOX12 using immunofluorescence. Most immunostains of ALOX12 and the mitochondrial marker exhibited colocalization, indicating that ALOX12 potentially participates in Ca^2+^-induced mitochondrial lipid peroxidation (Fig. 6D and Fig. S6B). In addition, the fluorescence intensity of ALOX12 was stronger after Ca^2+^ influx compared with that of the control and after pretreatment with an ALOX12 inhibitor and BAPTA-AM. The ALOX12 inhibitor, ML355, was used to investigate ALOX12 function in calcium overload and RSL3-induced ferroptosis. Pretreatment with ML355 prevented Ca^2+^-induced lipid peroxidation and cell injury (Fig. 6E and F and Fig. S6C). We further validated the function of ML355 in RSL3-triggered ferroptosis. Inhibition of ALOX12 attenuated lipid peroxidation and ferroptotic cell injury (Fig. 6G and H and Fig. S6C). Therefore, TRPM2-induced calcium accumulation and increased ALOX12 expression in mitochondria likely led to mitochondrial lipid peroxidation.

### Pharmacological inhibition of TRPM2 attenuates hepatic IR injury in vivo

**Fig. 7.**
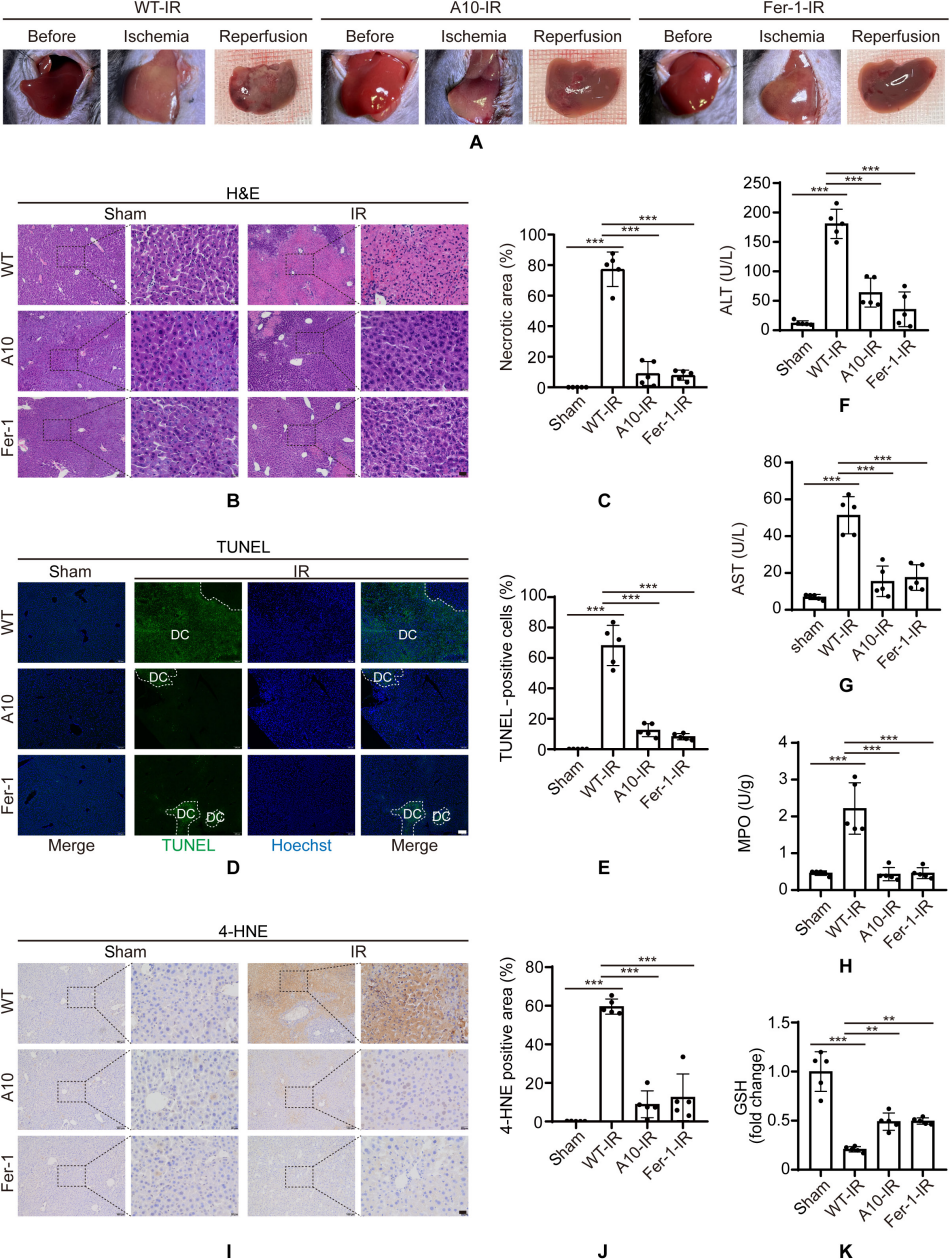
Inhibition of TRPM2 alleviates hepatic ischemia–reperfusion injury in vivo*.* (A) Representative images showing morphological changes in the liver at various stages from wild-type mice pretreated with phosphate-buffered saline, A10, or ferrostatin-1 for 60 min before ischemia, followed by 60-min partial hepatic ischemia, and 24-h reperfusion. (B) Representative hematoxylin and eosin staining and (C) quantification showing histopathological changes. Scale bars: 100 μm and 20 μm. (D) Representative terminal deoxynucleotidyl transferase-mediated dUTP nick-end labeling staining images and (E) quantification of liver specimens. Scale bars: 100 μm. (F and G) Mean serum alanine and aspartate aminotransferase levels in mice under indicated conditions. (H) Mean myeloperoxidase levels in liver samples from mice treated with indicated conditions. (I) Representative 4-hydroxy-2-nonenal immunostaining images and (J) quantification of liver sections. Scale bars: 100 μm and 20 μm. (K) Mean glutathione levels in liver samples from mice under indicated conditions using 3 to 5 mice for each group. All results are shown as mean ± standard deviation. *P < 0.05, **P < 0.01, ***P < 0.001. IR, ischemia–reperfusion; Fer-1, ferrostatin-1; DC, dying cells.

Finally, we verified the role of TRPM2-mediated ferroptosis in hepatic IR injury in vivo. A 60-min partial hepatic ischemia and 24-h reperfusion performed on the WT mice resulted in liver injury (Fig. 7A to G). Both A10 and Fer-1 treatment before exposure to IR not only dramatically attenuated liver necrosis (Fig. 7A to C) and hepatic cell death (Fig. 7D and E), but also significantly improved liver function by reducing ALT and AST levels (Fig. 7F and G). In addition, A10 and Fer-1 treatments reduced hepatic IR injury-induced neutrophil infiltration (Fig. 7H), alleviated lipid peroxidation by reducing 4-HNE levels (Fig. 7I and J), and enhanced antioxidant capacity by increasing GSH levels (Fig. 7K). Taken together, these results confirm that the inhibition of TRPM2 activity can ameliorate hepatic IR injury in vivo.

## Discussion

In this study, our data demonstrate that TRPM2-mediated ferroptosis plays an important role during hepatic IR injury. In particular, our results suggest that TRPM2-induced Ca^2+^ influx leads to mitochondrial calcium accumulation via the MCU complex and increases ALOX12 expression to promote mitochondrial lipid peroxidation and ferroptosis. Furthermore, using our previously developed TRPM2-specific inhibitor, A10 [42], we made the striking discovery that inhibition of TRPM2 significantly reduced ferroptosis and hepatic IR injury, suggesting that TRPM2 is a potential therapeutic target for hepatic IR injury induced by liver resection and transplantation.

Previous studies have indicated that both calcium overload and oxidative stress are accompanied by hepatic IR injury [61]. Accumulating evidence showed that TRPM2 is essentially involved in IR injury in many different types of tissues, such as the brain, heart, and kidneys [7]. For example, TRPM2 mediates brain IR injury by regulating autophagy [13] and zinc accumulation [15]. Moreover, TRPM2 mediates acute kidney injury via a mechanism that involves the activation of Rac Family Small GTPase 1 (RAC1), oxidative stress, and mitochondrial apoptotic pathways [12]. However, the role of TRPM2 in cardiac IR injury remains unclear. A previous study showed that exposure of *TRPM2* knockout mice to myocardial IR led to less infarction than that in WT mice [16]. However, another study showed that TRPM2 preserved mitochondrial function and protected against myocardial IR injury in WT mice [62]. The findings reported in our study show that TRPM2 mediates hepatic IR injury through a novel mechanism by regulating mitochondrial lipid peroxidation and ferroptosis. Mounting evidence indicates that ferroptosis is a major contributor to IR-associated cell death in various tissues owing to oxidative stress [63]. As a ROS sensor, TRPM2 can be activated during IR injury of multiple tissues. In future studies, it will be important to investigate whether TRPM2 mediates ferroptosis during IR injury of the brain, kidney and heart. In addition, further clarification is needed on whether TRPM2-mediated signaling pathways during IR injury in other tissues are involved in hepatic IR injury.

It is well known that hepatic IR injury is one of the major issues in liver transplantation. A recent study found that high ferritin levels in donors could serve as a risk predictor of liver dysfunction after liver transplantation [23]. Other evidence also suggests that a high-iron diet exacerbates hepatic IR injury, and in turn, depletion of the hepatic iron content by iron chelation and inhibition of ferroptosis significantly attenuates hepatic IR injury in mice [23]. In addition, another study showed that increased macrophage extracellular trap formation in macrophages aggravated iron overload, triggered hepatic ferroptosis, and promoted post-ischemic liver damage [64]. To date, 3 pathways, including GPX4 and iron and lipid metabolites, are required for ferroptosis [63]. Interestingly, mounting evidence suggests that calcium overload may be involved in ferroptosis, and a recent study reported that the calcium-permeable channel, TRPV4, was upregulated during ferroptosis [65]; however, the underlying mechanism is unknown. In our study, RNA sequencing analysis revealed that differentially expressed genes associated with TRPM2-induced hepatic IR injury were clustered in key pathways of ferroptosis, such as the oxidation reduction and GSH metabolic processes. As expected, our results showed that TRPM2 activation-induced ferroptosis resulted in hepatic IR injury both in vivo and in vitro. Mechanistically, TRPM2-mediated extracellular Ca^2+^ influx leads to lipid peroxidation, which further mitigates ferroptosis.

We further found that TRPM2-induced calcium influx was mainly increased in mitochondria via the MCU1 complex. Interestingly, we also found that the expression of the lipoxygenase ALOX12 was increased in outer mitochondrial membrane, which mainly induces phospholipid peroxidation during ferroptosis. Similarly, Rådmark and Samuelsson [60] purified ALOX5 from human leukocytes and found that calcium is required for its activity through in vitro reactions. Mittal et al. [59] reported that ALOX12 expression and activity in zebrafish are both Ca^2+^-dependent. Our results showed that increased intracellular calcium induced by TRPM2 activation triggered calcium accumulation in the mitochondria and increased ALOX12 expression promoted mitochondrial lipid peroxidation and ferroptosis. It will be interesting to uncover the relationship between mitochondrial calcium overload and ALOX12 activity during TRPM2-mediated hepatic IR injury in the future.

Although some evidence supports the role of mitochondria in ferroptosis [27,66], recent studies indicate that mitochondria play a central role in ferroptosis as the major intracellular organelles for ROS generation, iron homeostasis, and glutaminolysis [67,68]. The results exhibited in this study indicate that TRPM2-mediated Ca^2+^ influx leads to Ca^2+^ accumulation in mitochondria and may trigger ferroptosis by inducing mitochondrial lipid peroxidation. Accumulating evidence has shown that TRPM2 plays a key role in mitochondrial dysfunction in many pathological processes such as Alzheimer's disease, stroke, inflammation, and diabetes [69]. For example, TRPM2 can mediate zinc accumulation in mitochondria during oxidative stress in neurons and microglia [15,70]. Other studies have also reported that TRPM2 can regulate the Ca^2+^/CaMKII and Bcl-2 pathways [71,72]. Accordingly, previous studies have reported that different Ca^2+^ sources induce liver IR injury through mitofusin-2-mediated mitochondrial Ca^2+^ uptake, Bcl-2-dependent increase in Ca^2+^-induced apoptosis [73,74], and the Ca^2+^/CaMKII pathway that promotes the production of proinflammatory factors [75]. Further investigation is required to decipher whether such Ca^2+^-dependent downstream signaling pathways are involved in TRPM2-mediated hepatic IR injury.

This study showed that TRPM2 deficiency or pharmacological inhibition of TRPM2 alleviates hepatic IR injury in mice by suppressing ferroptosis. Mechanistically, TRPM2-mediated Ca^2+^ influx leads to accumulation of mitochondrial calcium via MCU1 and increases ALOX12 expression, which, in turn, promotes mitochondrial lipid peroxidation and ferroptosis. These findings uncover a novel role for TRPM2 in ferroptosis and provide mechanistic insights into the process by which ferroptosis induces hepatic IR injury. Pharmacological inhibition of TRPM2 activity prevented hepatic IR injury, suggesting that TRPM2 may be a potential drug target for hepatic IR injury.

## Materials and Methods

### Study design

In this study, the function of TRPM2 during hepatic IR was verified in a mouse model in WT and TRPM2 knockout mice. The mice treated or not treated with TRPM2 inhibitor were also detected. Five mice were used in each group. Then, HE staining, TUNEL staining, and serum markers, for example, ALT, AST, and MPO, were detected to evaluate the role of TRPM2 in hepatic IR. RNA sequencing was applied to explore the potential mechanism of TRPM2-mediated hepatic IR injury, and transcriptome data indicated that TRPM2 induced injury through mediating ferroptosis. Thus, the ferroptosis markers were detected in a cell and mouse model including mitochondrial morphology by electron microscope, lipid peroxidation through flow cytometry, MDA, 4-HNE by IHC staining, GPX4, and NRF2 through Western blot. To investigate the location or expression of downstream protein ALOX12, confocal laser scanning microscopy was used. Intracellular calcium was detected by using fluorescent probe via confocal imaging. The method and materials were demonstrated in detail as follows.

### Mouse model of liver IR

TRPM2^–/–^ mice were originally generated at the University of Leeds and later bred and housed at the Laboratory Animal Center of Zhejiang University (Zhejiang, China). The generation of TRPM2^–/–^ mice were reported in our previous study; briefly, embryonic stem cell clones lacking exons 17 and 18 of the *TRPM2* gene were injected into C57BL/6-derived blastocysts and primary TRPM2^–/–^ mice obtained. After several rounds of crossbreeding, homozygous transgenic mice were generated and validation was performed through PCR of genomic DNA [46]. In this study, male WT and TRPM2^–/–^ C57BL/6 mice (8 to 10 weeks old and taken from their littermates) were used. Mice were anesthetized by intraperitoneal injection of 60 mg/kg pentobarbital. Blood supply to the left lateral/median lobes of the liver was occluded using an atraumatic clip and ischemic lobes became pale after 1 to 2 min. The clip was removed after 1 h of ischemia, and the mice were placed in an animal incubator for 24 h to allow reperfusion. Mice were put on the heating pad, which was thermostatically controlled at 37 °C throughout the experiment [76,77]. Following reperfusion, the mice were sacrificed. Additionally, WT mice were sacrificed after 3-, 6-, 12-, 24-, and 48-h reperfusion, respectively. Their serum samples and liver tissues were collected. WT and TRPM2^–/–^ mice for the sham control experiment underwent the same procedures, but without vascular occlusion. Five mice were used for each group.

The TRPM2-specific inhibitor, A10 (6-bromo-8-methyl-2-(3-phenyl-1H-pyrazol-4-yl)-2,3-dihydroquinazolin-4(1H)-one), was designed and synthesized previously in our laboratory [42]. In our animal experiments, A10 was dissolved in sterile physiological saline containing 5% castor oil. WT mice were intravenously injected with 30 mg/kg A10 at 1 h before ischemia surgery, and then mice were sacrificed 24 h after reperfusion. As a positive control, WT mice were treated with 10 mg/kg Fer-1 (S7243; Selleck Chemicals, Houston, TX, USA), a ferroptosis inhibitor. Five mice were used for each group.

All procedures were applied based on the *Guide for the Care and Use of Laboratory Animals* of the National Institutes of Health and the Animal Welfare Act guidelines, and approved by the Zhejiang University Experimental Animal Ethics Committee.

### Histopathology and immunohistochemistry

The liver specimens were fixed in 4% paraformaldehyde overnight and then processed, embedded in paraffin, and sliced into 5-μm sections. Then, sections were dewaxed and rehydrated by processing with xylene and decreasing concentrations of ethanol. After washing in phosphate-buffered saline (PBS), sections were either subjected to H&E staining (Solarbio, Beijing, China) or immunostained. Necrotic areas were quantitatively measured using ImageJ (version 1.8.0), and the necrotic area/entire area was used to indicate necrosis. The production of 4-HNE by lipid peroxidation was examined using immunohistochemistry with anti-4-HNE antibodies (ab46565; Abcam, Cambridge, UK). Histopathological and immunostaining images were observed via a microscope (Olympus Model BX43; Tokyo, Japan) at 100× magnification and analyzed using ImageJ software. The 4-HNE-positive and entire areas were used to calculate the percentage of the 4-HNE-positive area.

### TUNEL staining

The TUNEL assay was used to detect death cell (Beyotime, Shanghai, China). Paraffin sections were dewaxed, rehydrated, and washed in PBS as described in the Mouse model of liver IR section, before incubation with DNase-free Protease K for 30 min at 37 °C. After washing in PBS, the sections were stained with TUNEL, and nuclei were stained by Hoechst (Beyotime). Fluorescence images were captured using a fluorescence microscope (Olympus Model BX53; Tokyo, Japan) at excitation wavelengths of 488 nm and 405 nm for TUNEL and Hoechst staining, respectively. TUNEL- and Hoechst-positive cells were used to calculate the percentage of TUNEL-positive cells.

### Transmission electron microscopy

TEM was conducted at the Research Center of Diagnostic Electron Microscopy of Zhejiang University School of Medicine. Liver tissue specimens were fixed with 3% glutaraldehyde, and after being post-fixed in 1% osmic acid for 2 h, tissues were dehydrated with gradient concentrations of acetone, infiltrated, and pre-embedded in LX-112 resin in propylene oxide (1:1) overnight. Thereafter, the specimens were embedded in pure LX-112 resin and polymerized at 60 °C for 3 days. Samples were sectioned at 50 nm and stained by using uranyl acetate and lead citrate. Mitochondrial morphology was examined using TEM (Tecnai G2 Spirit 120 kV; FEI, Czech Republic). Ferroptotic mitochondria were identified based on the morphological characteristics of shrinkage, increased membrane density, and reduction or loss of mitochondrial cristae.

### RNA extraction, sequencing, and analysis

RNA was extracted from the liver tissues of WT and TRPM2^–/–^ mice with or without hepatic IR injury using TRIzol (Invitrogen, Carlsbad, CA, USA). RNA sequencing was performed using an LC-BIO system (LC-BIO, Hangzhou, China). Three liver samples from each group were analyzed. Differentially expressed genes were selected using the DEseq2 R package with a Log_2_ ratio of ±1.0 and *P* < 0.05. Volcano plots were created, and GO pathway enrichment analyses and GSEA were analyzed using OmicStudio tools (www.omicstudio.cn/tool).

### Cell culture

Mouse hepatocyte AML12 cells were bought from the ATCC. They were cultured in Dulbecco’s Modified Eagle Medium/nutrient mixture F-12 (DMEM-F12; Meilun Bio, Dalian, China) with 10% fetal bovine serum, 100 U/ml penicillin/0.1 mg/ml streptomycin (Biosharp, Hefei, China), 1% insulin-transferrin-selenium (Thermo Fisher Scientific, Waltham, MA, USA), and 40 ng/ml dexamethasone (Beyotime). Human hepatocyte MIHA cells, kindly provided by Dr. J. R. Chowdhury (Albert Einstein College of Medicine, New York, NY, USA), were cultured in DMEM (Gibco, Shanghai, China) with 10% fetal bovine serum and 100 U/ml penicillin/0.1 mg/ml streptomycin. All cells were maintained in an incubator at 5% CO_2_ and 37 °C.

### Isolation of mouse primary hepatocytes

Primary hepatocytes were isolated from 8-week-old male WT and TRPM2^–/–^ mice. The mice were anesthetized with 60 mg/kg pentobarbital injected intraperitoneally, perfused through portal vein cannulation with 100 ml of Hanks’ balanced salt solution without Ca^2+^ and Mg^2+^ (HBSS; Meilun Bio) containing 0.5 mM EGTA (Beyotime), and preheated to 37 °C using a mini-osmotic pump (Longer, China) at a rate of 8 ml/min. Following this, 150 ml of HBSS containing 1 mM CaCl_2_ and 60 mg/L collagenase IV (Worthington, USA) was perfused via portal vein cannulation at a rate of 6 ml/min at 37 °C. The digested liver specimens were shredded and filtered through a 40-μm nylon mesh (Falcon, USA) to disperse the cells. Primary hepatocytes were collected by centrifugation at 50 × *g* at 4 °C for 2 min 4 times. Cell viability was assessed using trypan blue staining. Primary hepatocytes were cultured in DMEM supplemented with 10% fetal bovine serum and 100 U/ml penicillin/0.1 mg/ml streptomycin. Primary hepatocytes were seeded in 96-well plates coated with rat tail collagen type I (Sigma-Aldrich, MO, USA) at a density of 1 × 10^4^ cells per well for the cell viability assay or in 24-well collagen-coated plates at a density of 7 × 10^4^ cells per well for the lipid peroxidation assay.

### Oxygen and glucose deprivation/reperfusion

MIHA and AML12 cells were cultured in serum- and glucose-free DMEM or DMEM-F12 media and incubated under 5% CO_2_ and 95% N_2_ at 37 °C for 12 h. For reperfusion, MIHA and AML12 cells were incubated in fresh DMEM or DMEM-F12 media with 10% fetal bovine serum under normal culture conditions for 2 h, 4 h, 8 h, 12 h, and 24 h, respectively. In some experiments, cells were treated with 10 μM A10 or 5 μM Fer-1 during reperfusion [78]. Cells without OGD/R were used as the controls. Three independent experiments were performed.

### Cell viability and cytotoxicity assays

AML12 and MIHA cells were seeded at densities of 8 × 10^3^ and 2 × 10^4^ cells per well in 96-well plates, respectively, and incubated overnight. Primary hepatocytes were seeded overnight. Cells were treated with 10 μM A10 or 5 μM Fer-1 for 30 min at 37 °C during exposure to 10 μM RSL3 for 24 h. Cell counting kit-8 (APExBIO, USA) was used to detect the cell viability, and LDH release assays (Beyotime) were performed to investigate cell cytotoxicity. Three independent experiments were applied for each assay.

### Lipid peroxidation assays

AML12 cells, MIHA cells, and primary hepatocytes were seeded in plates, respectively. The next day, cells were treated with 10 μM A10 or 5 μM Fer-1, 0.1 μM ML355 (HY-12341; MCE, New Jersey, USA), or 10 μM MCU-i4 (HY-138620; MCE) for 30 min at 37 °C during exposure to 10 μM RSL3 for 24 h. The levels of lipid ROS were assessed using C11-BODIPY 581/591 (Thermo Fisher Scientific), a probe for lipid peroxidation. Briefly, 10 μM C11-BODIPY 581/591 was added into cell medium and incubated for 1 h at 37 °C. Fluorescence intensity was determined using flow cytometry and fluorescence microscopy. Fluorescence intensity was measured using a fluorescence microscope (Olympus Model BX53; Tokyo, Japan) with excitation wavelengths of 488 and 405 nm for C11-BODIPY 581/591 and Hoechst, respectively. Hoechst staining was used to stain nuclei. For quantification of fluorescence intensity, mean fluorescence intensity (MFI) was calculated from representative areas after subtraction of background MFI using ImageJ software version 1.53c. Five micrograph fields were evaluated for each specimen.

### Intracellular Ca^2+^ measurement

AML12 and MIHA cells were seeded in plates and incubated overnight. Thereafter, the culture medium was replaced with HBSS, and after adding 4 μM Cal-520 AM calcium dye (AAT Bioquest, California, USA) to bind intracellular calcium into the medium, cells were incubated at 37 °C for 60 min, as previously described [79]. After washing in PBS, the cells were treated with 500 μM H_2_O_2_, 2 mM CaCl_2_, 30 μM RSL3, 10 μM MCU-i4, and 10 μM A10 for 30 min. Fluorescence intensity was measured using a fluorescence microscope, where a stronger fluorescence intensity indicates a greater intracellular Ca^2+^ concentration. Cal-520 AM and Hoechst staining were measured after excitation at 488 and 405 nm, respectively. For quantification of fluorescence intensity, MFI was calculated from representative areas after subtraction of background MFI using ImageJ software. Five micrograph fields were evaluated for each specimen.

### Mitochondrial fluorescence measurement

For mitochondrial Ca^2+^ distribution measurements, Cal-520 AM and Rhod-2 AM were used. Cal-520 AM staining was performed as previously described in the Lipid peroxidation assays section. For mitochondria staining, 200 nM MitoTracker Deep Red FM was added into cells and incubated for 20 min at 37 °C (Thermo Fisher Scientific). Hoechst staining was used to stain nuclei.

To specifically detect mitochondrial Ca^2+^ influx, the cells were incubated in 2.5 μM Rhod-2 AM dye at 25 °C for 30 min in the dark [47–49]. Thereafter, cells were washed with PBS thrice and then incubated for an additional 30 min in HBSS containing 200 nM MitoTracker Deep Red at 37 °C to allow de-esterification of intercellular Rhod-2 AM and stain mitochondria. Afterward, cells were washed with PBS, and treated with 10 μM A10, 5 μM BAPTA-AM, 0.1 μM ML355, or 10 μM MCU-i4 for 30 min, followed by treatment with 500 μM H_2_O_2_, 2 mM CaCl_2_, or 30 μM RSL3 for 30 min in HBSS. Hoechst staining was used to stain nuclei.

For mitochondrial lipid peroxidation measurements, C11-BODIPY 581/591 staining was applied as in the Intracellular Ca^2+^ measurement section. MitoTracker Deep Red (200 nM) was used to stain the mitochondria and Hoechst staining was used to stain the nuclei.

Cal-520 AM, Rhod-2 AM, C11 BODIPY 581/591, MitoTracker Deep Red FM, and Hoechst dyes were excited at 488, 550, 488, 640, and 405 nm, respectively. Fluorescence signals were captured using a confocal microscope (Leica TCS SP8, Germany). Mitochondrial Ca^2+^ and lipid peroxidation were quantified relative to the mitochondrial content (the area of Rhod-2 or C11 BODIPY 581/591 co-localizing with MitoTracker). The MFI of Rhod-2 AM- or C11 BODIPY 581/591-stained mitochondrial content was calculated after subtraction of background MFI using ImageJ software. For each specimen, 5 to 10 micrograph fields were evaluated.

### Mitochondrial malondialdehyde measurement

A mitochondrial isolation kit (Beyotime) was used for mitochondrial isolation. Briefly, MIHA cells were pretreated with A10 (10 μM) or BAPTA-AM (5 μM) for 30 min, followed by treatment with 500 μM H_2_O_2_, 2 mM CaCl_2_, or 10 μM RSL3 for 8 h in DMEM with 10% fetal bovine serum. The cells were then collected and resuspended in 1,000 μl of PBS. A 100-μl cell suspension was used to measure the concentration of protein with a bicinchoninic acid assay (Meilun Bio). Other cell suspensions were centrifuged and resuspended in mitochondria isolation reagent with 0.25 mM PMSF at 4 °C for 10 min. After centrifugation at 600 × *g* for 10 min at 4 °C, the supernatant was collected, and precipitated mitochondria were obtained by centrifugation at 11,000 × *g* for 10 min at 4 °C. The MDA content of isolated mitochondria was measured using an MDA assay kit according to its instructions (Njjcbio, Nanjing, China).The MDA levels were exhibited as nmol/mg protein.

### Mitochondrial membrane potential assay

The JC-1 probes (Solarbio) were used to detect the mitochondrial membrane potential. AML12 and MIHA cells were seeded, respectively. After drug treatment, cells were digested and collected, and washed with PBS. Thereafter, cells were resuspended with medium and JC-1 probes at 37 °C. After 20 min, cells were analyzed using flow cytometry. Fluorescence at excitation wavelengths of 540 nm and 488 nm indicated the healthy and damaged mitochondria, respectively. Three independent experiments were performed.

### Western blotting

After drug treatment, total cell lysis of MIHA and AML12 cells was performed using RIPA lysis buffer (Beyotime). The protein concentration of each sample was quantified using a bicinchoninic acid assay. Protein samples (50 μg) were separated on 5% to 12% SDS-PAGE gels and transferred to polyvinylidene fluoride membranes (Millipore, Darmstadt, Germany). After blocking with 5% bovine serum albumin, membranes were incubated with anti-human/mouse Nrf2 (A0674; Abclonal, Wuhan, China), anti-human/mouse GPX4 (52455l; Cell Signaling Technology), anti-ALOX12 (ER1903-58; Huabio, Hangzhou, China), anti-MCU (ER1803-57; Huabio), anti-MICU1 (ER1803-99; Huabio), anti-Ferritin (ET1610-78, Huabio, Hangzhou, China), or anti-human/mouse β-actin (ab6276; Abcam) antibodies at a dilution of 1:1,000 at 4 °C overnight, respectively. After washing with Tris-buffered saline containing Tween, the membranes were incubated with secondary HRP-conjugated goat anti-rabbit IgG (ab6721; Abcam) or goat anti-mouse IgG (ab6728; Abcam) antibodies at a concentration of 1:1,000 at 25 °C. One hour later, membranes were washed with phosphate buffered saline with Tween-20, and proteins were visualized using enhanced chemiluminescence (FDbio, Hangzhou, China). Protein expression was analyzed according to the gray value using ImageJ software.

### Quantitative PCR

Total RNA (2.5 μg) in a total volume of 50 μl of buffer was reverse-transcribed using the Evo M-MLV PT Premix (Accurate Biotechnology, Hunan, China) following these conditions: 37 °C for 15 min, 85 °C for 5 s, and reactions performed in a thermal cycler (Bioer Technology, Hangzhou, China). Next, 10 ng of cDNA was used for amplification of target genes (Table S1) using quantitative PCR with the following conditions: 95 °C for 30 s, followed by 95 °C for 5 s, and 60 °C for 30 s for a total of 40 cycles, and performed in a QuantStudio 6 Flex device (Thermo Fisher Scientific). CT values (cycle numbers at which fluorescence reached the threshold) were used to quantify mRNA expression of the target genes. The housekeeping gene, *GAPDH*, was used as a cDNA quality control and to normalize the expression levels of target genes [ΔCt = Ct (gene of interest) − Ct (*GAPDH*)]. Differences in mRNA expression between the groups were expressed as 2^−ΔΔCt^ [ΔΔCt = ΔCt (experimental group) − ΔCt (control group)]. The primer sequences used are listed in Table S1.

### Additional assays

GSH levels in liver tissues were measured using a GSH assay kit (Njjcbio) according to the manufacturer’s instructions. Liver tissues were homogenized in 0.9% saline at a tissue weight (g)/solution volume (ml) ratio of 1:9. After centrifugation, the supernatant was used for assays. Serum ALT and AST activities for mice in each group were measured using ALT and AST assay kits (Njjcbio) according to the manufacturer’s instructions. Five samples were examined in each group. The myeloperoxidase activity of mice in each group was measured using a myeloperoxidase assay kit (Njjcbio) according to the manufacturer’s instructions. Samples were prepared in the same way as that of the GSH assay. Liver tissues were prepared in a suspension through a homogenate. Five samples were examined in each group.

### Data presentation and statistical analyses

Data are shown as mean ± standard deviation and were analyzed using GraphPad Prism version 8.0 (www.graphpad.com). One-way and two-way analyses of variance with Tukey’s post hoc test were used to compare 3 or more groups. Statistical significance was set at *P* < 0.05.

## Data Availability

All data supporting the findings of this study are available within the paper and its supplementary information.
