# Supplementary material for: TRPM2 Mediates Hepatic Ischemia–Reperfusion Injury via Ca2+-Induced Mitochondrial Lipid Peroxidation through Increasing ALOX12 Expression
Source: Research (Wash D C). 2023 May 31;6:0159. doi: 10.34133/research.0159 (PMC10232356; doi:10.34133/research.0159)
Supplement: Supplementary 1 — Figs. S1 to S6 Table S1 [file research.0159.f1.docx]

Supplementary Materials


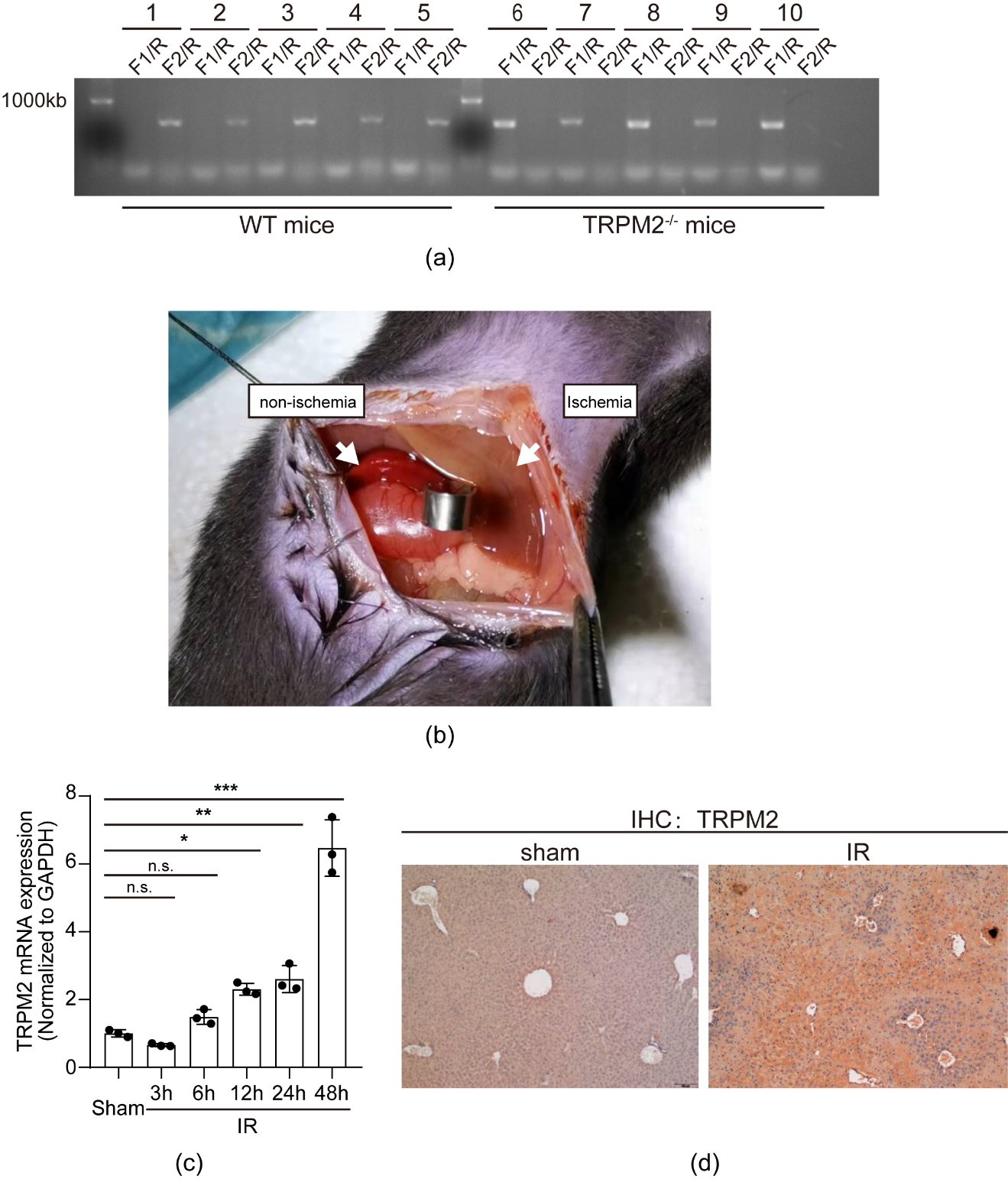


**Supplementary Figure 1. Identification of TRPM2^-/-^ mice and establishment of hepatic ischemia-reperfusion model. (a)** TRPM2^-/-^ mice were identified using PCR. Mouse embryonic stem cell clones carrying the mutated allele lacking exons 17 and 18 of the *TRPM2* gene and homozygous transgenic mice were obtained by several rounds of cross-breeding. TRPM2^-/-^ mice that express mutated alleles without exons 17 and 18 of the *TRPM2* gene were validated by PCR of genomic DNA. Forward primer 1 (F1) was designed for exons 17 and 18 and Forward primer 2 (F2) for exon 18 amplification assays. **(b)** Representative image of hepatic partial ischemia-reperfusion (IR). Blood supply to the left lateral/median lobes of the liver was occluded using an atraumatic clip. Left lateral/median lobes of the liver represented ischemia, while the right lobes represented non-ischemia. **(c)** The TRPM2 mRNA of liver tissues under sham-operated or subjected to 60 min of 70% partial warm ischemia, followed by 3, 6, 12, 24 h and 48h reperfusion. **(d)** The expression of TRPM2 protein in liver tissues during IR**.** n.s., no significance. **P* <0.05, ***P* <0.01, ****P* <0.001.


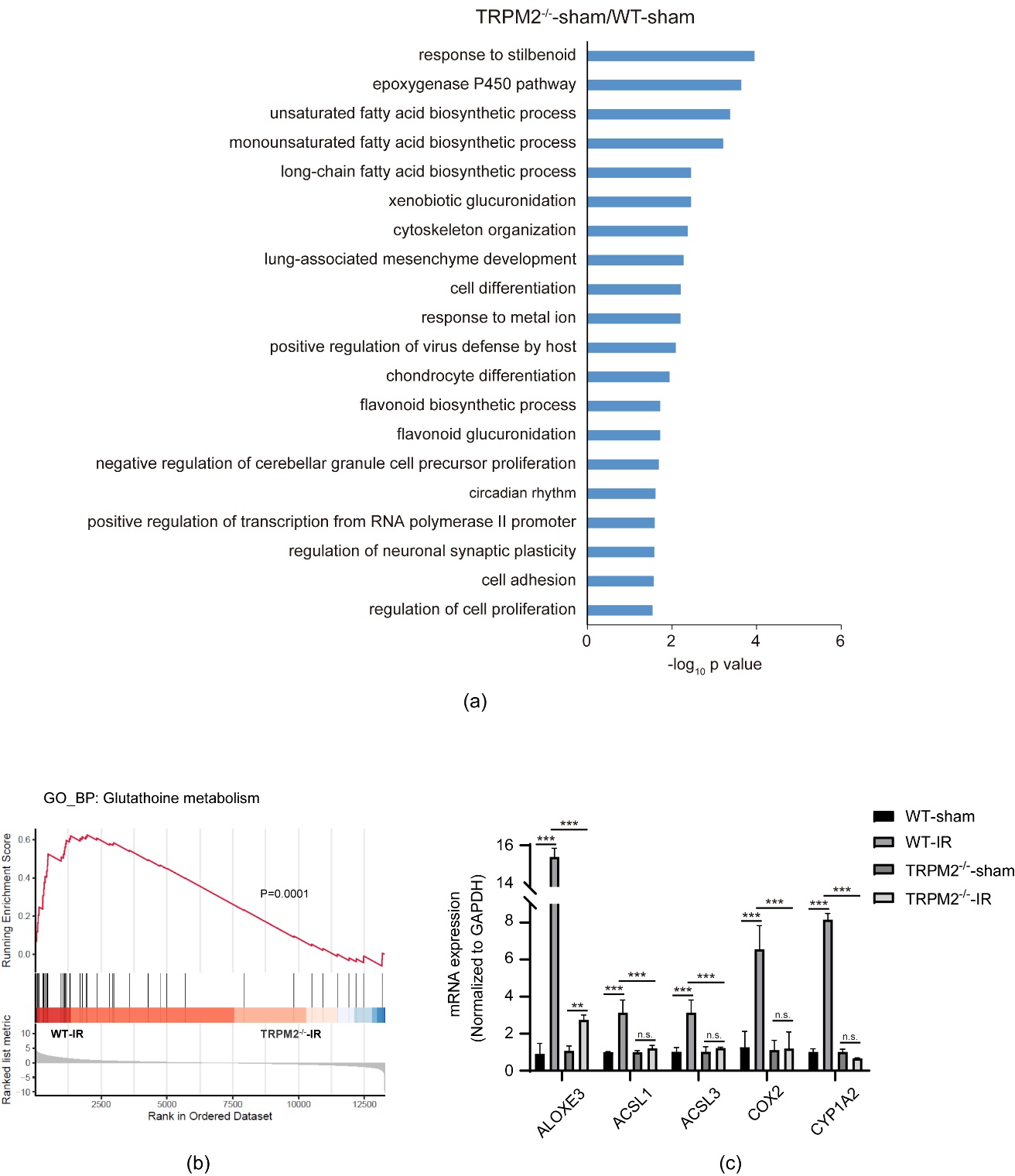


**Supplementary Figure 2. (a)** Gene ontology pathway enrichment analysis of deferentially expressed genes generated from TRPM2^–/–^ and wild-type (WT) mice that underwent a sham operation. **(b)** Gene Set Enrichment Analysis of glutathione metabolism in WT mice subjected to ischemia-reperfusion injury compared to that of TRPM2^–/–^ mice. (**c**) The mRNA expression of ferroptosis-related genes. n.s. = no significance, **P* <0.05, ***P* <0.01, ****P* <0.001.


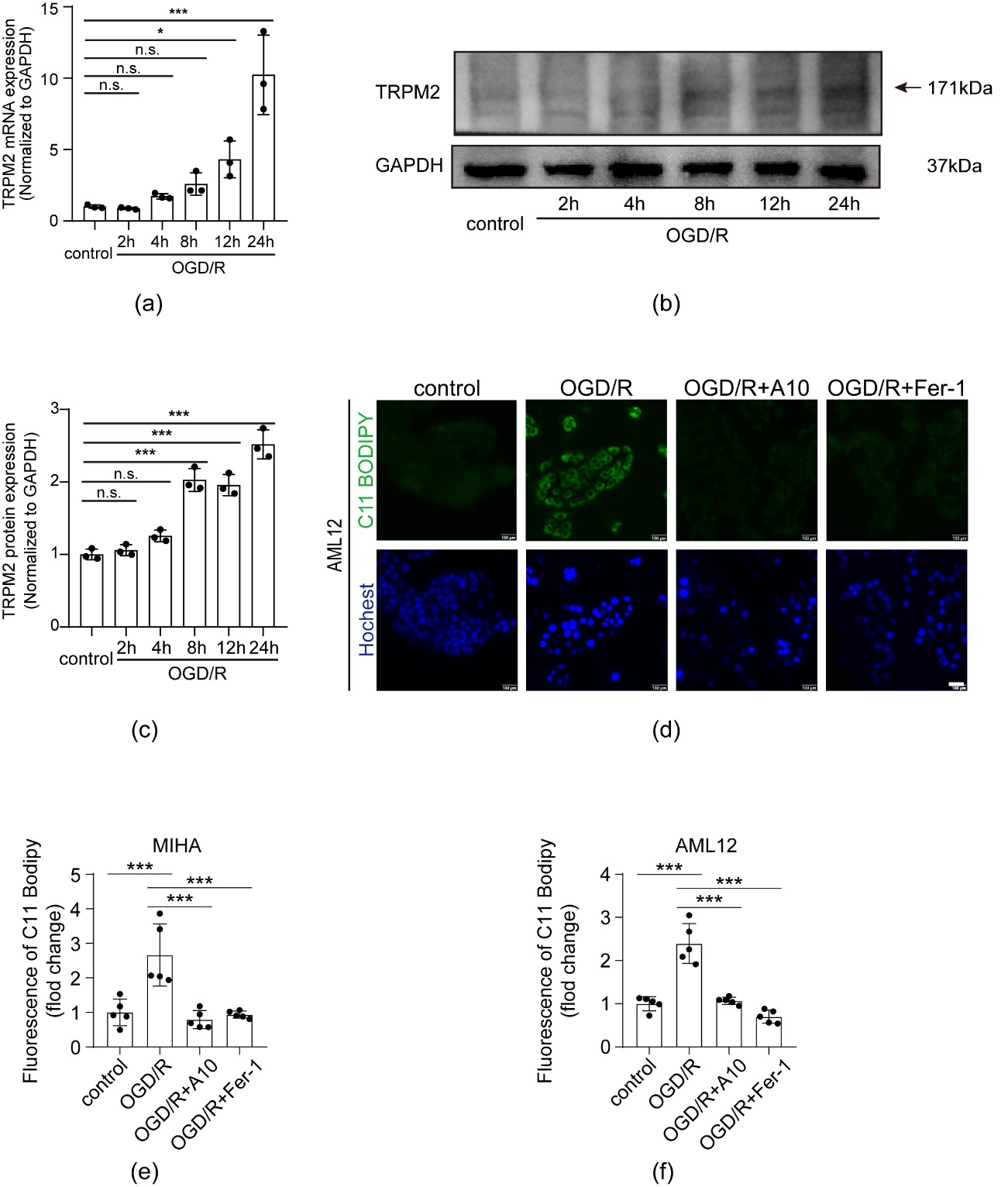


**Supplementary Figure 3.** **Inhibition of TRPM2 alleviates oxygen and glucose deprivation/reperfusion injury via reducing lipid peroxidation.** **(a)** The mRNA and (**b, c**) protein expression of TRPM2 in MIHA cell line under control or subjected to 12h of oxygen and glucose deprivation, followed by 2, 4, 8, 12 and 24 hours reperfusion. (**d**) Representative C11 BODIPY 581/591 staining (green) images and **(e)** quantification of lipid peroxidation levels in AML12 cells without or with exposure to OGD/R. Cells were counterstained with Hoechst (blue). Scale bar: 100 μm. (**f**) Quantification of C11 BODIPY 581/591 fluorescence showing the lipid peroxidation levels in MIHA cells without or with exposure to OGD/R in Figure 3f. All results are shown as mean ± standard deviation. n.s. = no significance, **P* <0.05, ***P* <0.01, ****P* <0.001. OGD/R, oxygen and glucose deprivation/reperfusion.


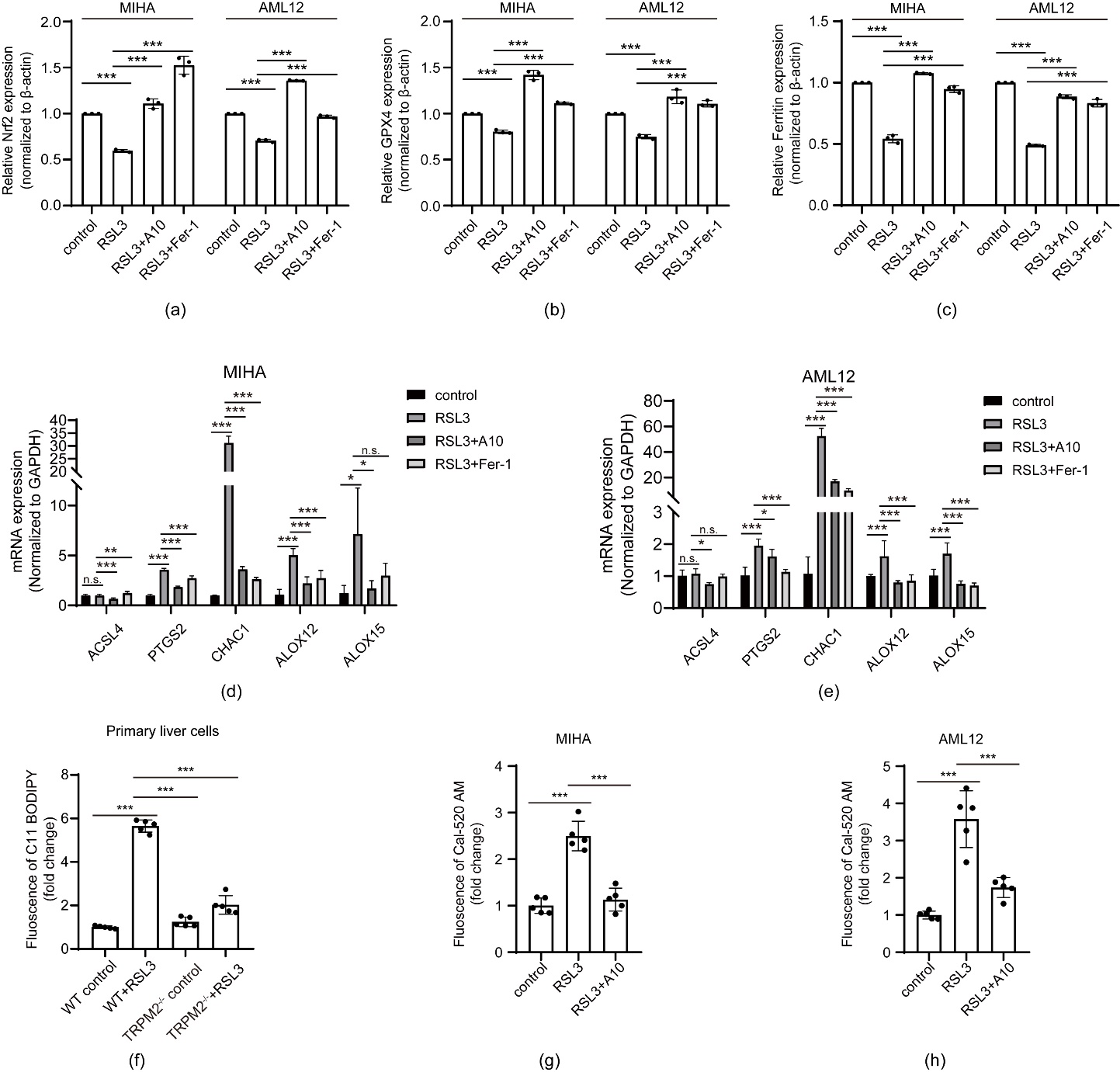


**Supplementary Figure 4. Quantification of images from Figure 4. (a, b, c)** Quantification of Nrf2, GPX4 and Ferritin protein expression for Figure 4j. (**d, e**) The mRNA expression of ferroptosis-related genes. **(f)** Quantification of C11 BODIPY 581/591 fluorescence for Figure 4k. **(g, h)** Quantification of Cal-520 AM fluorescence for Figures 4m and n. All results are shown as mean ± standard deviation. n.s. = no significance, **P* <0.05, ***P* <0.01, ****P* <0.001. n.s., no significance.


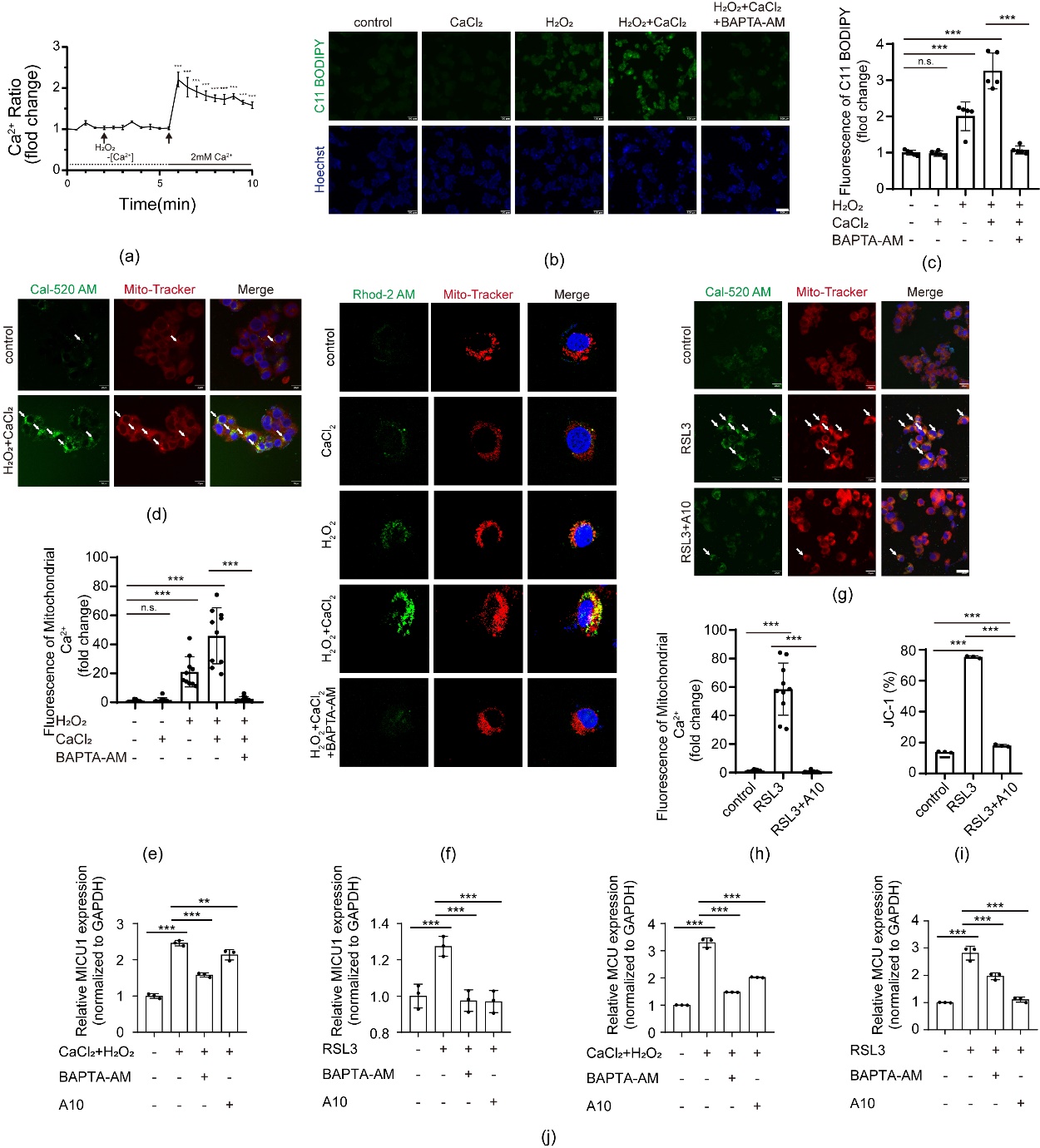


**Supplementary Figure 5. Calcium influx aggravates lipid peroxidation. (a)** Ca^2+^ mobilization assays for the MIHA liver cells. MIHA liver cells were loaded with Cal-520 AM and washed with PBS thrice before being resuspended in phosphate-buffered saline for flow cytometry. Thereafter, 500 μM H_2_O_2_ and 2 mM CaCl_2_ were added at 2 min and 5.5 min, respectively. **(b)** Representative C11 BODIPY 581/591 (green) staining images and **(c)** quantification showing lipid peroxidation levels. Cells were counterstained with Hoechst (blue). Scale bar: 100 μm. **(d)** Representative confocal micrographs showing intercellular Ca^2+^ with Cal-520 AM (green) staining and mitochondria with MitoTracker (red) staining. Cells were counterstained with Hoechst (blue). Scale bar: 20 μm. **(e)** Quantification of mitochondrial Ca^2+^ using Rhod-2 fluorescence from Figure 5b in the yellow area (the merged green and red channels). **(f)** Representative confocal micrographs of mitochondrial Ca^2+^ using Rhod-2 AM (green) staining and mitochondria with MitoTracker (red) staining for complementing Figure 5d. Scale bar: 5 μm. **(g)** Representative confocal micrographs showing intercellular Ca^2+^ with Cal-520 AM (green) staining and mitochondria with MitoTracker (red) staining under RSL3 treatment. Cells were counterstained with Hoechst (blue). Scale bar: 20 μm. **(h)** Quantification of mitochondrial Ca^2+^ from Figure 5e using Rhod-2 fluorescence in the yellow area. **(i)** Flow cytometry of mitochondrial membrane potential using JC-1 staining. **(j)** Quantification of MCU protein expression from Figure 5j**.** All results are shown as mean ± standard deviation. n.s. = no significance, **P* <0.05, ***P* <0.01, ****P* <0.001. n.s., no significance.


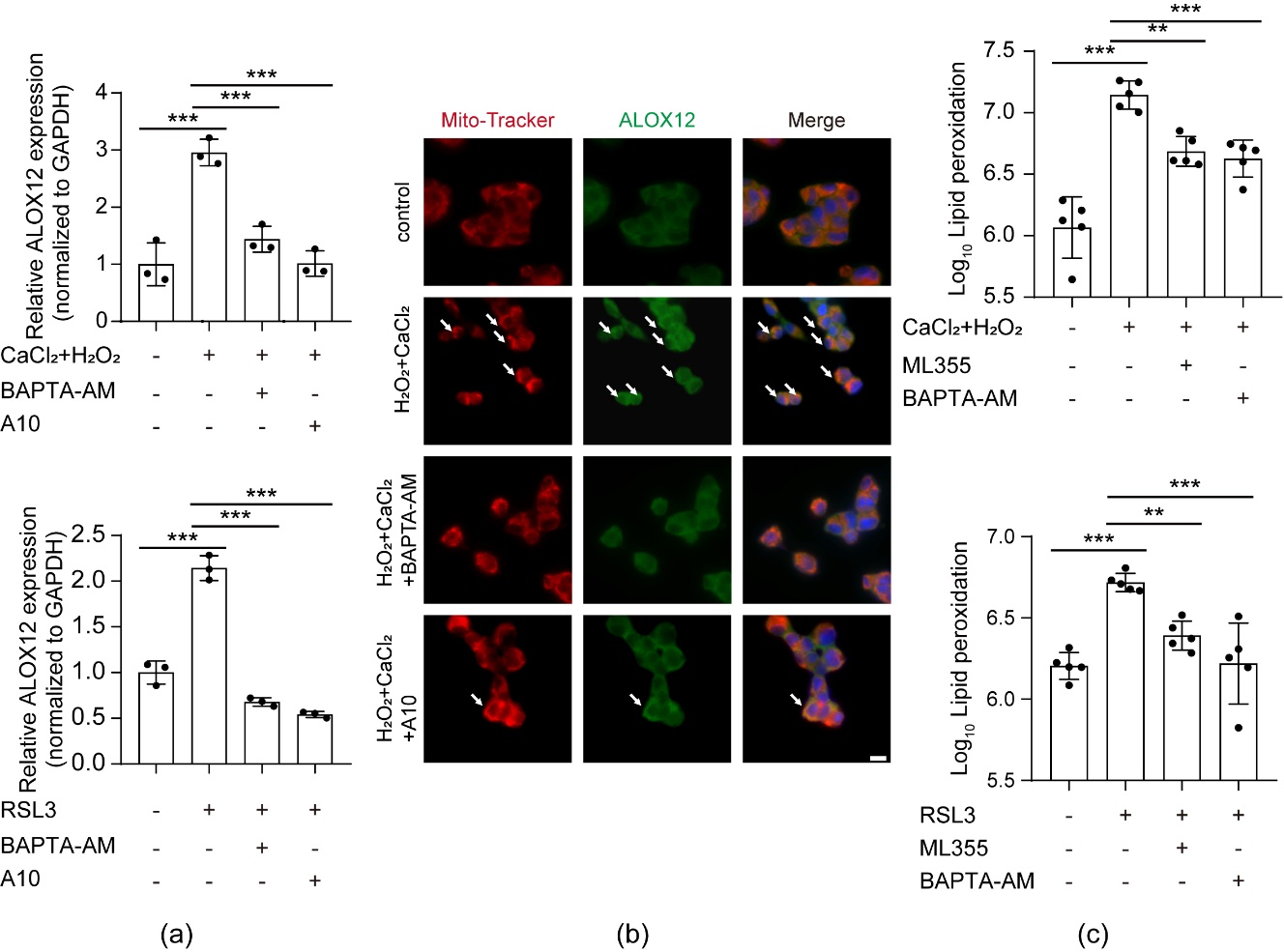


**Supplementary Figure 6. Quantification of images from Figure 6. (a)** Quantification of ALOX12 protein expression from Figure 6c. **(b)** Representative confocal micrographs of ALOX12 (green) staining and mitochondria with MitoTracker (red) staining for complementing Figure 6d. Scale bar: 10 μm. **(C)** Quantification of lipid peroxidation fluorescence from Figures 6d and f. All results are shown as mean ± standard deviation. ***P* <0.01, ****P* <0.001.

**Supplement Table 1.** List of primers used in qPCR analysis.

| Gene | Species | FORWARD Sequence (5’->3’) | REVERSE Sequence (5’->3’) |
| --- | --- | --- | --- |
| ALOX5 | Human | ACTGGCTGAATGACGACTGG | CAGGGGAACTCGATGTAGTCC |
| ALOX12 | Human | TCTGGAGATGGCCCTCAAAC | GAAGCTCTTCCATCCCCGAG |
| ALOX15 | Human | GGGGCAAGGAGACAGAACTC | CAGCTCTTCTTCCCGGTGTT |
| GAPDH | Human | TTGGTATCGTGGAAGGACTCA | TGTCATCATATTTGGCAGGTT |
| MCU | Human | AGGATCGGGGAATTGACAGAG | GTGTGGTGTATAGTTGCTGGAC |
| MICU1 | Human | AGAGCATCATTCGCTCCCAAA | GCAGTTTACGCTGAAATTCGAG |
| MICU2 | Human | GGACAGTGGCTAAAGTGGAGC | CATGAGGCGAGTGAAACCC |
| MICU3 | Human | AGGCGATTTCGTTTATTTGCTTC | GTGTTTCTGCGAGCATTTGATT |
| VDAC | Human | ACGTATGCCGATCTTGGCAAA | TCAGGCCGTACTCAGTCCATC |
| PTGS2 | Human | AGGTTTAGCAGCCACTGTAAC | GGGGTTTCGATCCAAACAAGC |
| CHAC1 | Human | GAACCCTGGTTACCTGGGC | CGCAGCAAGTATTCAAGGTTGT |
| TRPM2 | Human | GAGGACATCAGCAATAAGGCAG | ATGCGAGGGTGGTTACTGGA |
| ACSL1 | Mouse | TGACCTCTCCATGCAGTCAG | AGCCTATGCACTCAGCCAGT |
| ACSL3 | Mouse | AACCACGTATCTTCAACACCATC | AGTCCGGTTTGGAACTGACAG |
| ACSL4 | Mouse | CTCACCATTATATTGCTGCCTGT | TCTCTTTGCCATAGCGTTTTTCT |
| ALOXE3 | Mouse | ACCAGTGGATCGATGGCTAC | TGAAAGCTGCTGACATCCAC |
| COX2 | Mouse | TTCAACACACTCTATCACTGGC | AGAAGCGTTTGCGGTACTCAT |
| CYPA1 | Mouse | CACTAACGGCAAGAGCATGA | TCTGAAGCTTGCTGACGAGA |
| GAPDH | Mouse | CCATGACAACTTTGGCATTG | CCTGCTTCACCACCTTCTTG |
| PTGS2 | Mouse | TATGCCACCATCTGGCTTCG | CAATGTTGAAGGTGTCGGGC |
| CHAC1 | Mouse | CCAAGCCCTGTGGATTTTCG | AACTTGGTATGCCACACCCC |
| TRPM2 | Mouse | CTACGATCCTCCCTTTTACACC | ATCCACGACGTTGTAACTGATC |
